# Early-life infections and childhood cancer risk: a nationwide cohort study of 2 million children

**DOI:** 10.1186/s12885-026-15927-1

**Published:** 2026-04-13

**Authors:** Seoyeon Oh, Surabhi Shah, Jongmin Oh, Eun Sun Yoo, Ji Hyen Lee, Byungmi Kim, Bohyun Park, Logan G. Spector, Eun Mi Jung, Eunhee Ha, Jin-Hong Kim, Yi-Jun Kim

**Affiliations:** 1 Department of Occupational and Environmental Medicine, College of Medicine, Ewha Womans University, Seoul, Republic of Korea; 2 Institute of Ewha-SCL for Environmental Health (IESEH), College of Medicine, Ewha Womans University, Seoul, Republic of Korea; 3 Department of Human Systems Medicine, College of Medicine, Seoul National University, Seoul, Republic of Korea; 4 Department of Pediatrics, Ewha Womans University College of Medicine, Seoul, Republic of Korea; 5 Division of Cancer Prevention, National Cancer Control Institute, National Cancer Center, Goyang, Republic of Korea; 6 Department of Cancer Control and Population Health, Graduate School of Cancer Science and Policy, National Cancer Center, Goyang, Republic of Korea; 7 Division of Epidemiology and Clinical Research, Department of Pediatrics, University of Minnesota, Minneapolis, MN USA; 8 Masonic Cancer Center, University of Minnesota, Minneapolis, MN USA; 9 Division of Cancer Epidemiology and Genetics, National Cancer Institute, National Institutes of Health, Rockville, MD USA; 10 Graduate Program in System Health Science and Engineering, College of Medicine, Ewha Womans University, Seoul, Republic of Korea; 11 Center for RNA Research, Institute for Basic Science, Seoul, Republic of Korea; 12 Department of Biological Sciences, College of Natural Sciences, Seoul National University, Seoul, Republic of Korea; 13 Interdisciplinary Program in Bioinformatics, Seoul National University, Seoul, Republic of Korea

**Keywords:** Communicable diseases, Infection control, Medical oncology, Pediatric

## Abstract

**Background:**

The relationship between early-life infections and childhood cancer risk remains unclear despite its importance for risk stratification and clinical surveillance.

**Methods:**

We conducted a nationwide population-based cohort study using the South Korean National Health Information Database. Children born between January 1, 2002, and December 31, 2006, were followed through December 31, 2019. Two analytic cohorts were defined: Cohort 1 (*n* = 1,996,174) evaluated the number of infection episodes during ages 0–4 years, with a restricted analysis of infections occurring between ages 2–4 years; Cohort 2 (*n* = 1,999,065) evaluated infections during ages 0–1 year. Infection episodes were defined using healthcare visits with relevant ICD-10 diagnostic codes. Incident childhood cancer was ascertained from claims records. Multivariable Cox models estimated hazard ratios (HRs) adjusting for sex, residence, income quintile, and birth year.

**Results:**

Hazard ratios (HRs) were estimated relative to the lowest infection frequency category for each infectious disease. Upper respiratory tract infections, pneumonia, and influenza were not associated with increased cancer risk. At ages 0–1 year, risk increased with 2–4 lower respiratory infections (HR 1.06, 95% CI 1.00–1.12), ≥ 2 enteric infections (HR 1.11, 95% CI 1.01–1.22), ≥ 1 urinary infection (HR 1.21, 95% CI 1.09–1.33), and ≥ 2 mycoses (HR 1.19, 95% CI 1.03–1.38). At ages 2–4 years, risk remained elevated for ≥ 1 urinary infection (HR 1.13, 95% CI 1.02–1.25) and for skin infections (HR 1.09, 95% CI 1.02–1.17 for one episode; HR 1.13, 95% CI 1.03–1.24 for ≥ 2 episodes). Hospitalization showed a dose–response pattern: for ages 0–4 years, HRs were 1.07 (95% CI 1.00–1.14) for one admission, 1.17 (95% CI 1.04–1.32) for two, and 1.30 (95% CI 1.14–1.47) for ≥ 3; for ages 0–1 year, 1.07 (95% CI 1.00–1.15) for one and 1.22 (95% CI 1.05–1.41) for ≥ 2; for ages 2–4 years, 1.12 (95% CI 1.04–1.21) for one and 1.18 (95% CI 1.03–1.36) for ≥ 2. Limitations include potential outcome and exposure misclassification inherent to claims data, unmeasured confounding (e.g., genetic susceptibility, environmental factors), and use of hospitalization as a proxy for infection severity.

**Conclusions:**

Infections managed in outpatient settings were not associated with increased cancer risk, whereas infections requiring hospitalization—used as a proxy for more severe infections—were associated with higher cancer incidence and showed a dose–response pattern. Infection severity and frequency may be relevant to childhood cancer development and risk stratification.

**Supplementary Information:**

The online version contains supplementary material available at 10.1186/s12885-026-15927-1.

## Background

Childhood cancer, a leading cause of child mortality globally, is a substantial public health burden [1, 2]. Epidemiological data have indicated a steady rise in pediatric cancer incidence over recent decades, at an estimated annual increase of approximately 0.7% in the United States [1, 3]. Although this upward trend may be partly explained by enhanced diagnosis and improved cancer registration, environmental factors may also play a role [4–6]. Unlike adult cancers, the etiology of most pediatric malignancies is poorly understood because they lack well-defined environmental or lifestyle exposure [7]. Recent studies have also highlighted the potential contribution of inherited genetic susceptibility and germline mutations to the development of certain childhood cancers [8]. 

Among potential environmental factors, early-life infections have attracted considerable attention because of their critical influence on immune system maturation during infancy and early childhood [8–12]. A prevailing hypothesis is that early-life microbial exposures may condition the immune system to guard against premalignant development [13–16]. Therefore, insufficient exposure to infections during early childhood may leave the immune system inadequately primed, increasing susceptibility to aberrant inflammatory responses upon later infection and elevating cancer risk [14–16]. In addition, early-life antibiotic exposure may alter the developing gut microbiome, which plays a critical role in immune system maturation and may further influence susceptibility to immune-related diseases, including cancer [17]. 

Alternative evidence suggests that infection-triggered chronic inflammation and immune dysregulation might predispose to pediatric cancers [17–19]. Repeated infection episodes might promote genomic instability, oxidative stress, and immune dysregulation, which contribute to oncogenesis [20, 21]. Several studies have demonstrated a positive association between medically attended infections in early childhood and increased hematological and solid tumor incidence in children, suggesting that rather than inhibit carcinogenesis, infection-driven immune perturbation may facilitate it in some contexts [17–19].

These conflicting findings highlight the complexity of determining the precise relationship between early-life infections and pediatric cancer risk [22, 23]. Inconsistent infection exposure definition and quantification across studies is a major barrier to the clarification of this issue. Specifically, robust infection characterization, including clearly defined type, severity, timing, and infectious episode frequency, is essential but currently lacking. Moreover, many previous investigations utilized retrospective case–control designs, which are vulnerable to recall and selection bias [8, 9, 17–19, 24]. Additionally, infection exposure is frequently inferred from proxies like daycare attendance, birth month, season, and birth order, which may not accurately reflect infection history [9, 24].

Here, we undertook a nationwide population-based cohort study using data from South Korea’s National Health Insurance Service (NHIS), which covers approximately 98% of the national population, to obtain comprehensive insight into this relationship. This study leveraged high-resolution clinical records to systematically investigate associations between medically documented infections occurring in the first five years of life and the subsequent childhood cancer risk with the aim of clarifying the complex role of early-life infections in pediatric cancer etiology and ultimately guiding preventive and early intervention strategies.

## Methods

### Data source

This study was conducted using data from the National Health Insurance Database of South Korea’s NHIS, covering approximately 98% of the South Korean population [25]. The National Health Insurance Database includes detailed demographic, healthcare utilization, diagnosis, medication, health screening, and mortality data [26]. This study utilized demographic, death records, and medical claims data from 2002 to 2019 for children born between 1 January 2002 and 31 December 2006, representing birth cohorts that were followed from birth through early childhood. These children were subsequently observed during their early childhood period (ages 0–4 years).

### Study design

**Fig. 1:**
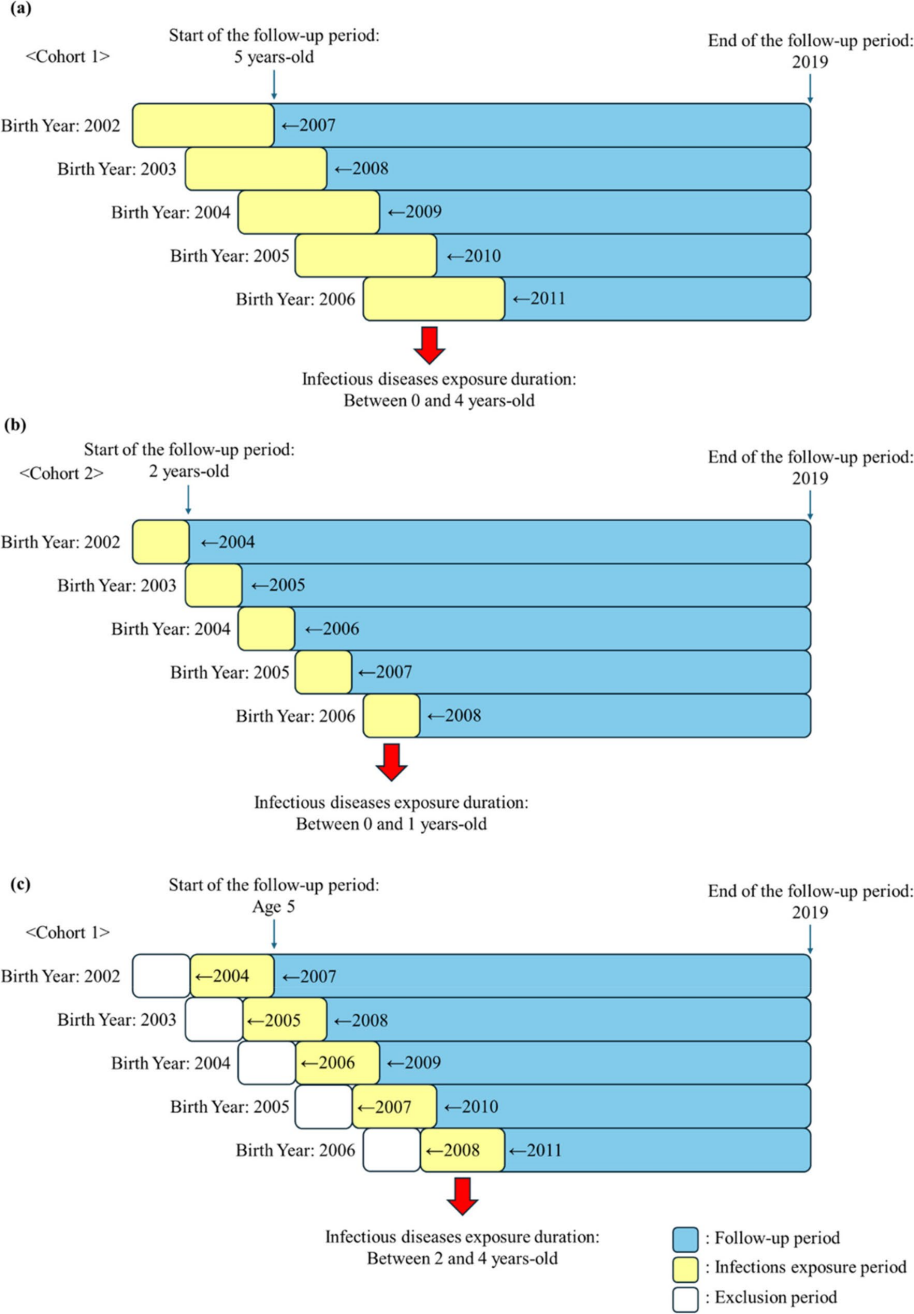
Overview of the two cohort designs from 2002 to 2019. **a** Cohort 1: infection exposure during ages 0–4 years. **b** Cohort 2: infection exposure during ages 0–1 year. **c** Secondary analysis within cohort 1 with the exposure window restricted to ages 2–4 years

Two cohort designs were used to evaluate the association between early childhood infections and childhood cancer risk across different age periods. The use of two cohort designs allowed us to assess infectious disease exposure during distinct stages of early childhood and to examine whether the timing of infection exposure influences subsequent cancer risk. The exposure windows were defined based on differences in immune system development during early childhood, as immune responses during the first two years of life are still influenced by neonatal immune characteristics, including reliance on maternally derived antibodies and an immature adaptive immune system, whereas immune function becomes more mature after infancy and early toddlerhood. Figure 1 is a study design overview. To evaluate the impact of infection exposure during the first five years of life on subsequent cancer risk, cohort 1 was comprised of all children born between 1 January, 2002, and 31 December, 2006, and survived to age five years (the 0–4-year-old group). The first five years of life were the exposure window. Follow-up began at each child’s fifth birthday and continued until the earliest of cancer diagnosis, death, or 31 December 2019 (Fig. 1a). Cohort 2, which included those who survived to two years old (the 0–1-year-old group) in the same birth cohorts (2002–2006), was used to assess the effect of infection exposure in the first two years of life on later cancer development. Follow-up commenced at each child’s second birthday and extended until cancer diagnosis, death, or 31 December, 2019 (Fig. 1b). Cohort 1 (exposure window: ages 2–5 years, the 2–4-year-old group) was used to evaluate the effect of infection exposure in the three years after the first two years of life. Follow-up began once each child reached the age of five years and continued until the earliest of cancer diagnosis, death, or 31 December 2019 (Fig. 1c). This design ensured that infection exposures during early childhood (ages 0–4 years) were assessed before the start of the cancer risk observation period, thereby minimizing potential reverse causation due to infections occurring during the preclinical phase of cancer.

### Exposures: infectious diseases

This study defined exposure as the frequency of outpatient visits or hospitalizations because of infectious diseases. The following infectious disease categories were selected: upper respiratory tract infections (URTIs), influenza, pneumonia, lower respiratory tract infections (LRTIs), unknown fever, enteric infections, ear infections, eye infections, urinary tract infections, skin infections, mycoses, herpes, chickenpox, other viral infections, other bacterial infections, and gastrointestinal tract infections. Infectious diseases were identified using International Classification of Diseases, 10th Revision, codes (Supplementary Table S1). Although pneumonia may overlap conceptually with LRTIs, we classified pneumonia separately from other LRTIs in this study according to the underlying diagnostic coding framework (pneumonia: J12-J18, LRTI: J20-J22) (Supplementary table S1). This approach was chosen to preserve the predefined code structure and improve the interpretability of the exposure categories. The frequency of each infectious disease was determined using diagnostic records in the medical claims table. Multiple diagnostic records of the same infectious disease within a 14-day window were considered one episode, whereas records separated by more than 14 days were treated as distinct episodes. A similar approach was applied regardless of the specific disease category to define the total frequency of outpatient visits or hospitalizations for all 16 infectious disease categories combined.

The frequency of outpatient visits or hospitalization because of each infectious disease was categorized into discrete groups based on the distribution of visit counts for each disease. For URTIs and LRTIs, frequency groups were defined based on the annual average number of visits during the exposure period. For all other infectious diseases, frequency groups were determined based on the total number of visits throughout the exposure period.

Because the frequency distribution of infection episodes differed substantially across infectious disease categories and across exposure windows, frequency groups were defined separately for each infection and exposure period based on the observed distribution of episode counts within the respective exposure window. For highly prevalent infections (e.g., upper and lower respiratory tract infections), where having no episodes during certain exposure windows was relatively uncommon, the lowest observed frequency category rather than strictly zero episodes was used as the reference group to ensure adequate sample size and stable risk estimation.

### Outcomes: cancers

This study’s primary outcome was cancer incidence, which was identified based on diagnostic records. Cancer outcomes were evaluated using both broader diagnostic groups and more specific subtypes, including lymphoid and hematopoietic cancer, leukemia, lymphoid leukemia, myeloid leukemia, lymphoma, Hodgkin lymphoma, non-Hodgkin lymphoma, central nervous system cancer, brain cancer, bone cancer, soft tissue sarcoma, renal cancer, liver cancer, or other cancers. To present both overall and subtype-specific associations, each cancer category was analyzed separately in a distinct outcome-specific model. The International Classification of Diseases, 10th Revision, codes used to define each cancer category are provided in Supplementary Table S2.

### Covariates

Sex, residence, income, and year of birth were included as covariates to account for potential confounding by demographic and socioeconomic factors. Residence classification was based on the area of residence at birth and was grouped as metropolitan, non-metropolitan, or “unknown” (missing data). Income level was determined based on insurance premium percentiles in the year of birth and was categorized into 17th − 20th, 14th − 16th, 11th − 13th, 8th − 10th, and 0–7th percentiles, or as unknown. Higher insurance premium percentiles are generally associated with higher income levels. The year of birth was treated as a categorical variable classified by individual years from 2002 to 2006. In addition to adjusting for these variables as potential confounders, we also examined their distributions and their associations with childhood cancer risk.

### Statistical analyses

Descriptive statistics for Cohorts 1 and 2 were summarized separately, and frequencies and percentages were reported for categorical variables, including sex, residence, income level, and birth year. Chi-square tests were used to assess baseline characteristics differences between children who developed cancer during follow-up and those who did not.

The models were adjusted for covariates, including sex, area of residence, household income, and year of birth. Because children could experience multiple infectious diseases during the exposure window, other infectious disease variables were simultaneously included in the multivariable Cox proportional hazards models to account for potential confounding by correlated or co-occurring infections. Before fitting the multivariable Cox proportional hazards (PH) models, the generalized variance inflation factor was used to assess multicollinearity among covariates. All covariates included in the final analyzes had generalized variance inflation factor values of < 2, indicating negligible multicollinearity. Multivariable Cox PH regression models were then fitted to estimate hazard ratios (HRs) and 95% confidence intervals (CIs) for cancer incidence based on infectious disease frequency categories. Cox PH model regression analyzes were performed separately based on hospital utilization type (combined hospitalizations and outpatient visits, hospitalizations only, and outpatient visits only) and defined age windows of infectious disease exposure (ages 0–4, 0–1, and 2–4 years). Cox PH models were used to analyze overall cancer risk based on the combined visit frequency for any infectious disease across different hospital utilization types and age windows. Subgroup analyzes by cancer type were also conducted to evaluate the cancer risk associated with the visit frequency for any infectious disease, stratified by hospital utilization type and age windows.

All statistical analyzes were conducted using R version 4.0.3 (R Foundation for Statistical Computing, Vienna, Austria) and SAS version 7.1 (SAS Institute Inc., Cary, NC, USA). This cohort study followed the STROBE guidelines.

## Results

**Fig. 2:**
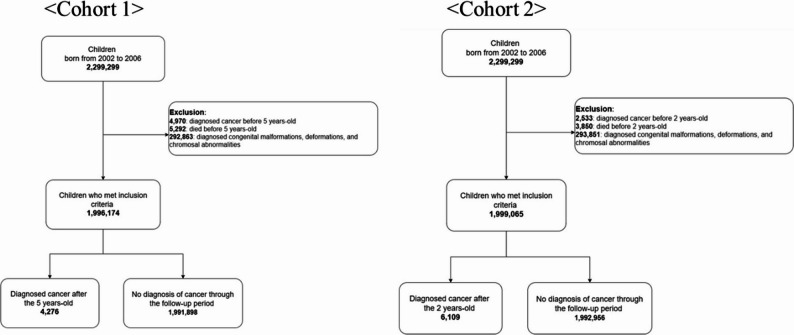
Flowchart of cohort 1 study subjects and cohort 2 study subjects. Because exclusion criteria were applied sequentially, the number of exclusions at each step may differ between cohort designs

This study used two distinct cohorts defined by the age at which follow-up began. In Cohort 1, follow-up started at the age of five years and continued through 2019 and two exposure windows were defined, (1) from birth to just before the age of five years (0–4 years), and (2) from the age of two years to just before the age of five years (2–4 years). In Cohort 2, follow-up began at the age of two years and continued through 2019, and exposure was restricted to the first two years of life (0–1 years, Fig. 2). Children with congenital malformations, deformations, or chromosomal abnormalities (e.g., trisomy 21), which are known to be associated with an increased risk of certain childhood cancers, were excluded from the study population [27].

Cohort 1 had 2,299,299 children born between 2002 and 2006. After excluding those diagnosed with cancer (4,970), those who died before the age of five (5,292), and those diagnosed with congenital malformations, deformations, or chromosomal abnormalities between 2002 and 2019 (292,863), 1,996,174 children remained, and of these, 4,276 were diagnosed with cancer after the age of five.

For Cohort 2, the same birth cohort (2002–2006) yielded an initial population from which those diagnosed with cancer (2,533), those who died before the age of two (3,850), and those with congenital malformations, deformations, or chromosomal abnormalities in 2002–2019 (293,851) were excluded, leaving 1,999,065 children, and of these, 6,109 were diagnosed with cancer after the age of two.

Baseline characteristics, including covariates and hospital utilization frequency, were compared between the cancer and non-cancer groups in Cohorts 1 and 2 using Chi-squared tests. Significant differences with respect to residence, income level, and year of birth were observed between the two groups in Cohort 1, while significant differences were found in residence and year of birth in Cohort 2 (Table S3).

**Fig. 3:**
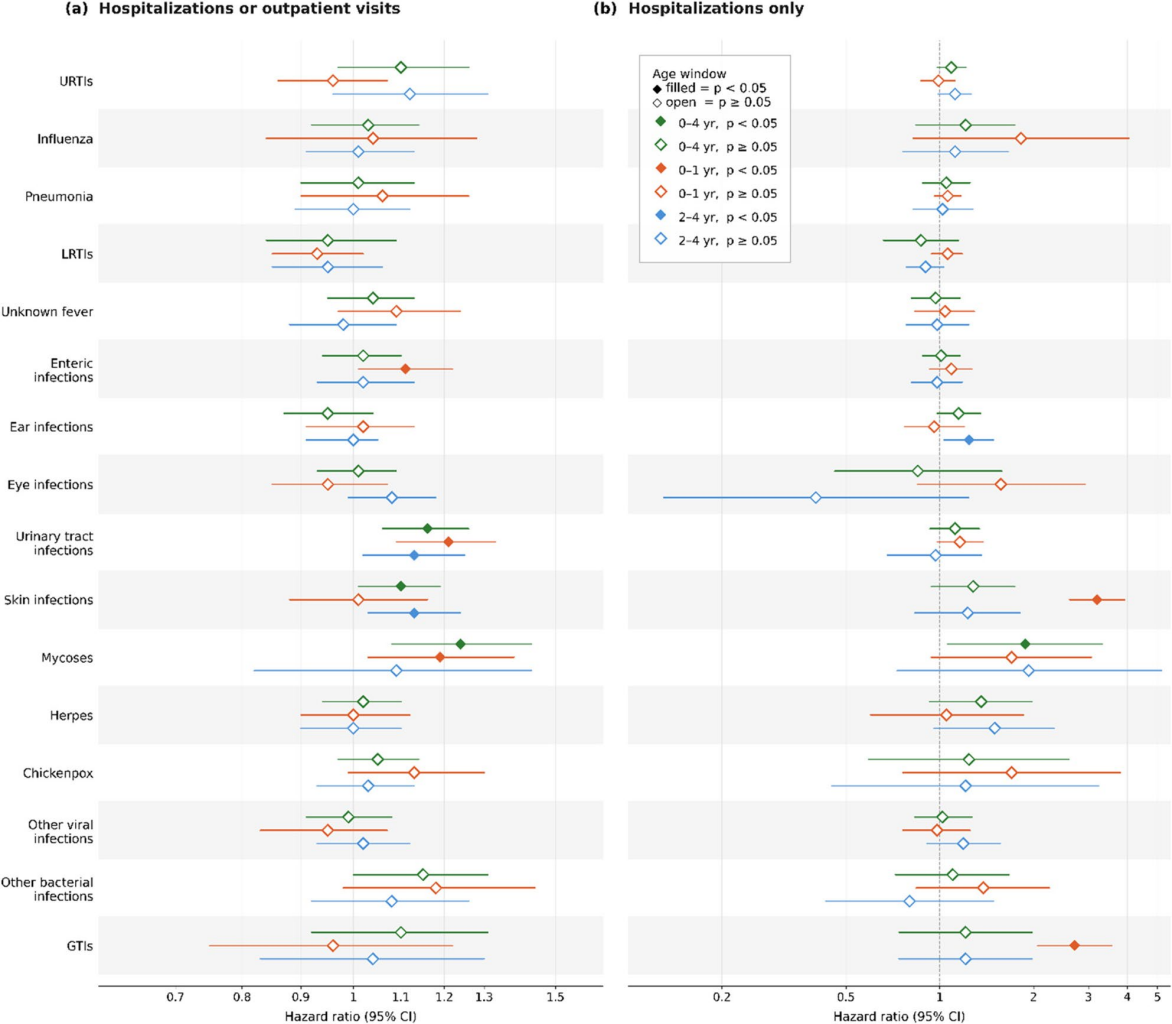
Forest plots of adjusted hazard ratios for childhood cancer incidence associated with early-life infectious disease exposures, corresponding to Tables 1 and 2. For each infection type, the highest-exposure stratum is plotted. **a** Hospitalizations or outpatient visits (Table 1). **b** Hospitalizations only (Table 2). The x-axis scale in panel (**b**) is wider than in panel (**a**) owing to broader confidence intervals from smaller exposed numbers. Abbreviations. CI, confidence interval; GTIs, gastrointestinal tract infections; HR, hazard ratio; LRTIs, lower respiratory tract infections; URTIs, upper respiratory tract infections

We examined the association between the frequency of outpatient visits and hospitalizations for infectious diseases during early childhood (age: 0–4 years) and subsequent cancer risk from the age of five years onwards (Fig. 3a, Table 1). Hazard ratios were estimated using the lowest frequency category for each infectious disease as the reference group. No significant association was found between upper respiratory tract infections, pneumonia, or influenza and subsequent cancer risk. However, individuals with at least one episode of urinary tract infection (HR: 1.16, 95% CI: 1.06–1.26), skin infection for one episode (HR: 1.08, 95% CI: 1.01–1.16), skin infection for ≥ 2 episodes (HR: 1.10, 95% CI: 1.01–1.19), or other bacterial infections (HR: 1.15, 95% CI: 1.00–1.31) exhibited a significantly elevated cancer risk. Although a single mycoses episode was not associated with a statistically significant increase in cancer risk, individuals with ≥ 2 episodes showed a significantly increased risk (approximately 24%; HR: 1.24, 95% CI: 1.08–1.43).

**Table 1:** Hazard ratios for cancer incidence based on hospital visit frequency (hospitalizations or outpatient visits) for each infectious disease type during three exposure windows (age: 0–4, 0–1, and 2–4 years).This analyses were conducted using two cohort designs with different exposure windows: cohort 1 evaluated infection exposure during ages 0–4 years, while cohort 2 evaluated exposure during ages 0–1 years. In addition, cohort 1 was further analyzed by separating the exposure window into 0–1 years and 2–4 years to examine potential differences in infection timing. P values indicate the statistical significance of hazard ratios estimated from Cox proportional hazards models

0–4 years old			0–1 years old			2–4 years old		
Infectious diseases, episodes	Number (%)	Hazard ratio (95% CI)	Infectious diseases, episodes	Number (%)	Hazard ratio (95% CI)	Infectious diseases, episodes	Number (%)	Hazard ratio (95% CI)
URTIs			URTIs			URTIs		
< 2/year	221,250 (11.08)	Reference	≤ 2/year	675,815 (33.81)	Reference	< 2/year	238,233 (11.93)	Reference
≥ 2 & <3/year	298,583 (14.96)	1.09 (0.96–1.24)	> 2 & ≤4/year	777,880 (38.91)	1.00 (0.95–1.07)	≥ 2 & <4/year	526,420 (26.37)	1.08 (0.97–1.21)
≥ 3 & <4/year	415,682 (20.82)	1.01 (0.90–1.14)	> 4 & ≤6/year	421,127 (21.07)	0.98 (0.92–1.05)	≥ 4 & <6/year	655,541 (32.84)	1.07 (0.96–1.20)
≥ 4 & <5/year	437,877 (21.94)	1.06 (0.94–1.20)	> 6/year	124,243 (6.22)	0.96 (0.86–1.07)	≥ 6 & <8/year	452,434 (22.67)	1.11 (0.98–1.24)
≥ 5 & <6/year	344,634 (17.26)	1.07 (0.94–1.21)				≥ 8/year	123,546 (6.19)	1.12 (0.96–1.31)
≥ 6/year	278,148 (13.93)	1.10 (0.97–1.26)						
Influenza			Influenza			Influenza		
0	1,762,451 (88.29)	Reference	0	1,973,135 (98.70)	Reference	0	1,781,433 (89.24)	Reference
≥ 1	233,723 (11.71)	1.03 (0.92–1.14)	≥ 1	25,930 (1.30)	1.04 (0.84–1.28)	≥ 1	214,741 (10.76)	1.01 (0.91–1.13)
Pneumonia			Pneumonia			Pneumonia		
0	777,210 (38.93)	Reference	0	1,391,200 (69.59)	Reference	0	1,007,637 (50.48)	Reference
1	520,486 (26.07)	1.00 (0.93–1.08)	1	383,277 (19.17)	0.95 (0.89–1.02)	1	467,247 (23.41)	1.05 (0.98–1.13)
2–4	511,021 (25.60)	0.99 (0.91–1.07)	2–3	177,658 (8.89)	1.05 (0.95–1.15)	2–3	339,829 (17.02)	1.04 (0.95–1.13)
≥ 5	187,457 (9.39)	1.01 (0.90–1.13)	≥ 4	46,930 (2.35)	1.06 (0.90–1.26)	≥ 4	181,461 (9.09)	1.00 (0.89–1.12)
LRTIs			LRTIs			LRTIs		
< 1/year	258,931 (12.97)	Reference	< 2/year	1,114,406 (55.75)	Reference	< 2/year	598,745 (29.99)	Reference
≥ 1 & <2/year	438,364 (21.96)	1.07 (0.96–1.19)	**≥ 2 & <4/year**	**630**,**390 (31.53)**	**1.06 (1.00–1.12)**	≥ 2 & <4/year	696,685 (34.90)	0.94 (0.87–1.02)
≥ 2 & <3/year	483,923 (24.24)	1.06 (0.95–1.18)	≥ 4/year	254,269 (12.72)	0.93 (0.85–1.02)	≥ 4 & <6/year	481,853 (24.14)	1.01 (0.93–1.11)
≥ 3 & <4/year	392,510 (19.66)	1.09 (0.97–1.22)				≥ 6/year	218,891 (10.97)	0.95 (0.85–1.06)
≥ 4 & <5/year	249,275 (12.49)	1.09 (0.96–1.24)						
≥ 5/year	173,171 (8.68)	0.95 (0.96–1.24)						
Unknown fever			Unknown fever			Unknown fever		
0	1,124,792 (56.35)	Reference	0	1,558,629 (77.97)	Reference	0	1,364,948 (68.38)	Reference
1	558,915 (28.00)	1.04 (0.97–1.12)	1	351,452 (17.58)	1.02 (0.96–1.09)	1	434,606 (21.77)	1.05 (0.97–1.13)
≥ 2	312,467 (15.65)	1.04 (0.95–1.13)	≥ 2	88,984 (4.45)	1.09 (0.97–1.24)	≥ 2	196,620 (9.85)	0.98 (0.88–1.09)
Enteric infections			Enteric infections			Enteric infections		
0	1,028,770 (51.54)	Reference	0	1,444,574 (72.26)	Reference	0	1,313,356 (65.79)	Reference
1	547,772 (27.44)	1.02 (0.95–1.09)	1	390,752 (19.55)	1.01 (0.95–1.08)	1	440,253 (22.05)	0.99 (0.92–1.06)
≥ 2	419,632 (21.02)	1.02 (0.94–1.10)	**≥ 2**	**163**,**739 (8.19)**	**1.11 (1.01–1.22)**	≥ 2	242,565 (12.15)	1.02 (0.93–1.13)
Ear infections			Ear infections			Ear infections		
0	359,343 (18.00)	Reference	0	1,019,922 (51.02)	Reference	0	550,893 (27.60)	Reference
1–2	674,638 (33.80)	0.92 (0.85–1.01)	1–2	653,650 (32.70)	0.98 (0.93–1.04)	1–2	727,504 (36.44)	0.96 (0.89–1.03)
3–4	362,840 (18.18)	0.98 (0.89–1.09)	3–4	193,796 (9.69)	0.97 (0.88–1.06)	3–4	323,186 (16.19)	1.02 (0.93–1.13)
≥ 5	599,353 (30.03)	0.95 (0.87–1.04)	≥ 5	131,697 (6.59)	1.02 (0.91–1.13)	≥ 5	394,591 (19.77)	1.00 (0.91–1.05)
Eye infections			Eye infections			Eye infections		
0	832,105 (41.68)	Reference	0	1,484,162 (74.24)	Reference	0	1,067,165 (53.46)	Reference
1	692,898 (34.71)	0.96 (0.89–1.03)	1	405,008 (20.26)	1.00 (0.94–1.06)	1	616,661 (30.89)	0.98 (0.91–1.05)
≥ 2	471,171 (23.60)	1.01 (0.93–1.09)	≥ 2	109,895 (5.50)	0.95 (0.85–1.07)	≥ 2	312,348 (15.65)	1.08 (0.99–1.18)
Urinary tract infections			Urinary tract infections			Urinary tract infections		
0	1,715,870 (85.96)	Reference	0	1,875,953 (93.84)	Reference	0	1,816,213 (90.98)	Reference
** ≥ 1**	**280**,**304 (14.04)**	**1.16 (1.06–1.26)**	**≥ 1**	**123**,**112 (6.16)**	**1.21 (1.09–1.33)**	**≥ 1**	**179**,**961 (9.02)**	**1.13 (1.02–1.25)**
Skin infections			Skin infections			Skin infections		
0	1,250,719 (47.63)	Reference	0	1,586,177 (79.35)	Reference	0	1,155,172 (57.87)	Reference
** 1**	**650**,**918 (32.61)**	**1.08 (1.01–1.16)**	1	335,295 (16.77)	0.99 (0.93–1.06)	**1**	**561**,**043 (28.11)**	**1.09 (1.02–1.17)**
** ≥ 2**	**94**,**537 (19.76)**	**1.10 (1.01–1.19)**	≥ 2	77,593 (3.88)	1.01 (0.88–1.16)	**≥ 2**	**279**,**959 (14.02)**	**1.13 (1.03–1.24)**
Mycoses			Mycoses			Mycoses		
0	1,576,147 (78.96)	Reference	0	1,710,352 (85.56)	Reference	0	1,833,381 (91.87)	Reference
1	332,899 (16.68)	1.02 (0.94–1.11)	1	234,704 (11.74)	1.04 (0.96–1.12)	1	138,614 (6.94)	1.05 (0.93–1.18)
** ≥ 2**	**87**,**128 (4.36)**	**1.24 (1.08–1.43)**	**≥ 2**	**54**,**009 (2.70)**	**1.19 (1.03–1.38)**	≥ 2	23,729 (1.19)	1.09 (0.82–1.43)
Herpes			Herpes			Herpes		
0	1,668,182 (83.57)	Reference	0	1,885,758 (94.33)	Reference	0	1,753,714 (87.85)	Reference
≥ 1	327,992 (16.43)	1.02 (0.94–1.10)	≥ 1	113,307 (5.67)	1.00 (0.90–1.12)	≥ 1	242,460 (12.15)	1.00 (0.90–1.10)
Chickenpox			Chickenpox			Chickenpox		
0	1,680,612 (84.19)	Reference	0	1,937,601 (96.93)	Reference	0	1,737,531 (87.04)	Reference
≥ 1	315,562 (15.81)	1.05 (0.97–1.14)	≥ 1	61,464 (3.07)	1.13 (0.99–1.30)	≥ 1	258,643 (12.96)	1.03 (0.93–1.13)
Other viral infections			Other viral infections			Other viral infections		
0	955,334 (47.86)	Reference	0	1,497,360 (74.90)	Reference	0	957,117 (47.95)	Reference
1	647,084 (32.42)	1.00 (0.93–1.07)	1	402,112 (20.12)	1.04 (0.97–1.10)	1	647,828 (32.45)	1.02 (0.94–1.09)
≥ 2	393,756 (19.73)	0.99 (0.91–1.08)	≥ 2	99,593 (4.98)	0.95 (0.83–1.07)	≥ 2	391,229 (19.60)	1.02 (0.93–1.12)
Other bacterial infections			Other bacterial infections			Other bacterial infections		
0	1,904,558 (95.41)	Reference	0	1,969,697 (98.53)	Reference	0	1,926,978 (96.53)	Reference
** ≥ 1**	**91**,**616 (4.59)**	**1.15 (1.00–1.31)**	≥ 1	29,368 (1.47)	1.18 (0.98–1.44)	≥ 1	69,196 (3.47)	1.08 (0.92–1.26)
GTIs			GTIs			GTIs		
0	1,941,340 (97.25)	Reference	0	1,977,548 (98.92)	Reference	0	1,960,675 (98.22)	Reference
≥ 1	54,834 (2.75)	1.10 (0.92–1.31)	≥ 1	21,517 (1.08)	0.96 (0.75–1.22)	≥ 1	35,499 (1.78)	1.04 (0.83–1.30)
Sex			Sex			Sex		
Male	1,020,895 (51.14)	Reference	Male	1,022,558 (51.15)	Reference	Male	1,020,895 (51.14)	Reference
** Female**	**975**,**279 (48.86)**	**1.06 (1.00–1.13)**	Female	976,507 (48.85)	0.97 (0.93–1.02)	**Female**	**975**,**279 (48.86)**	**1.06 (1.00–1.13)**
Residence			Residence			Residence		
Metropolitan	1,092,913 (54.75)	Reference	Metropolitan	1,094,387 (54.74)	Reference	Metropolitan	1,092,913 (54.75)	Reference
** Others**	**870**,**302 (43.60)**	**1.12 (1.05–1.19)**	**Others**	**871**,**658 (43.60)**	**1.13 (1.07–1.19)**	**Others**	**870**,**302 (43.60)**	**1.12 (1.05–1.19)**
Unknown	32,959 (1.65)	1.20 (0.96–1.50)	**Unknown**	**33**,**020 (1.65)**	**1.21 (1.01–1.46)**	Unknown	32,959 (1.65)	1.19 (0.95–1.49)
Income (percentile)			Income (percentile)			Income (percentile)		
17th − 20th	295,168 (14.79)	Reference	17th − 20th	295,550 (14.78)	Reference	17th − 20th	295,168 (14.79)	Reference
14th − 16th	420,121 (21.05)	0.94 (0.84–1.04)	14th − 16th	420,725 (21.05)	1.00 (0.91–1.09)	14th − 16th	420,121 (21.05)	0.94 (0.85–1.04)
11th − 13th	452,242 (22.66)	1.03 (0.93–1.14)	11th − 13th	452,889 (22.66)	1.04 (0.95–1.13)	11th − 13th	452,242 (22.66)	1.03 (0.94–1.14)
8th − 10th	367,586 (18.41)	0.93 (0.83–1.03)	8th − 10th	368,124 (18.41)	0.98 (0.89–1.07)	8th − 10th	367,586 (18.41)	0.93 (0.83–1.03)
0–7th	417,207 (20.90)	1.03 (0.93–1.14)	0–7th	417,878 (20.90)	1.06 (0.97–1.16)	0–7th	417,207 (20.90)	1.03 (0.93–1.14)
Unknown	43,850 (2.20)	1.04 (0.84–1.28)	Unknown	43,899 (2.20)	0.97 (0.80–1.16)	Unknown	43,850 (2.20)	1.04 (0.84–1.29)
Birth year			Birth year			Birth year		
2002	431,264 (21.60)	Reference	2002	431,989 (21.61)	Reference	2002	431,264 (21.60)	Reference
2003	426,789 (21.38)	0.97 (0.89–1.06)	2003	427,419 (21.38)	0.95 (0.88–1.02)	2003	426,789 (21.38)	0.97 (0.89–1.06)
2004	407,730 (20.43)	1.06 (0.96–1.16)	2004	408,321 (20.43)	0.99 (0.91–1.06)	2004	407,730 (20.43)	1.06 (0.96–1.16)
2005	372,142 (18.64)	0.96 (0.87–1.07)	2005	372,650 (18.64)	0.90 (0.83–0.98)	2005	372,142 (18.64)	0.96 (0.87–1.07)
2006	358,249 (17.95)	0.95 (0.84–1.06)	2006	358,686 (17.94)	0.87 (0.79–0.95)	2006	358,249 (17.95)	0.95 (0.85–1.07)

Statistically significant (*p* < 0.05) hazard ratios are indicated in bold

*CI* confidence interval, *URTI* upper respiratory tract infection, *LRTI* lower respiratory tract infection, *GTI* gastrointestinal tract infection

To examine how infection timing during childhood modifies cancer risk, we stratified early childhood by birth through ages 0–1 years and 2–4 years, and re-analyzed infectious disease frequency in each subgroup. During ages 0–1, only children who experienced 2–4 LRTI episodes per year (HR: 1.06, 95% CI: 1.00–1.12), those with ≥ 2 enteric infection episodes (HR: 1.11, 95% CI: 1.01–1.22), those with ≥ 1 urinary tract infection episode (HR: 1.21, 95% CI: 1.09–1.33), and those with ≥ 2 mycoses episodes (HR: 1.19, 95% CI: 1.03–1.38) showed a statistically significant increase in subsequent cancer risk. No evidence of infection having a protective effect against cancer was observed in this 0–1 year window. Between the ages of 2 and 4, urinary tract infections and skin infections remained significantly linked to increased cancer risk. Specifically, a single urinary tract infection episode during this period was associated with a 13% higher cancer risk (HR: 1.13, 95% CI: 1.02–1.25). One skin infection episode conferred a 9% risk increase (HR: 1.09, 95% CI: 1.02–1.17), which rose to 13% among those with ≥ 2 episodes (HR: 1.13, 95% CI: 1.03–1.24). Female sex and residence outside metropolitan areas were associated with an increased risk of childhood cancer, whereas household income and birth year were not associated with cancer incidence. In summary, URTI, influenza, and pneumonia were not associated with cancer risk in the 0–1 or 2–4 years of age windows, whereas severe or recurrent infections, presumably indicating impaired immunity, were associated with increased cancer incidence.

The association between hospitalization frequency for infectious diseases and subsequent cancer risk is presented in Fig. 3b and Table 2. Because infection-related hospitalizations were much less frequent than total infection episodes including outpatient visits, the exposure frequency categories in Table 2 were defined separately based on the distribution of hospitalization episodes. Over the 0–4-year period, only hospitalizations for mycoses were linked to a significant cancer risk increase (HR: 1.88, 95% CI: 1.06–3.32). In the 0–1-year age group, children hospitalized at least once for skin infections showed a markedly higher cancer risk (HR: 3.20, 95% CI: 2.61–3.92), as did those hospitalized for gastrointestinal infections (HR: 2.71, 95% CI: 2.06–3.57). Among children aged 2–4 years, hospitalizations for ear infections were significantly associated with increased cancer risk (24% higher risk in those hospitalized at least once (HR: 1.24, 95% CI: 1.03–1.49). Female sex and residence outside metropolitan areas remained associated with an increased risk of childhood cancer, whereas household income and birth year were not associated with cancer incidence.

**Table 2:** Cancer incidence hazard ratios based on hospital visit frequency (hospitalization only) by infectious disease type during three exposure windows (age: 0–4, 0–1, and 2–4 years). For the 2–4 years old exposure window analysis, the same cohort was retained as the 0–4 years old exposure window analysis, but only infectious disease exposures occurring between ages 2 and 4 years were considered

0–4 years old			0–1 years old			2–4 years old		
Infectious diseases, episodes	Number (%)	Hazard ratio (95% CI)	Infectious diseases, episodes	Number (%)	Hazard ratio (95% CI)	Infectious diseases, episodes	Number (%)	Hazard ratio (95% CI)
URTIs			URTIs			URTIs		
0	1,773,988 (88.87)	Reference	0	1,911,789 (95.63)	Reference	0	1,842,916 (92.32)	Reference
1	193,036 (9.67)	1.09 (0.98–1.21)	≥ 1	87,276 (4.37)	0.99 (0.87–1.12)	≥ 1	153,258 (7.68)	1.12 (0.99–1.26)
≥ 2	29,150 (1.46)	0.94 (0.71–1.25)						
Influenza			Influenza			Influenza		
0	1,982,876 (99.33)	Reference	0	1,998,047 (99.95)	Reference	0	1,983,842 (99.38)	Reference
≥ 1	13,298 (0.67)	1.21 (0.84–1.74)	≥ 1	1,018 (0.05)	1.82 (0.82–4.05)	≥ 1	12,332 (0.62)	1.12 (0.76–1.66)
Pneumonia			Pneumonia			Pneumonia		
0	1,771,021 (81.81)	Reference	0	1,837,585 (91.92)	Reference	0	1,749,427 (87.64)	Reference
1	193,476 (14.53)	1.05 (0.96–1.15)	1	142,836 (7.15)	1.06 (0.96–1.17)	1	204,626 (10.25)	1.10 (0.99–1.22)
≥ 2	31,677 (3.67)	1.05 (0.88–1.25)	≥ 2	18,644 (0.93)	1.02 (0.78–1.34)	≥ 2	42,121 (2.11)	1.02 (0.82–1.28)
LRTIs			LRTIs			LRTIs		
0	1,771,021 (88.72)	Reference	0	1,878,782 (93.98)	Reference	0	1,871,410 (93.75)	Reference
1	193,476 (9.69)	0.98 (0.88–1.10)	≥ 1	120,283 (6.02)	1.06 (0.94–1.18)	≥ 1	124,764 (6.25)	0.90 (0.78–1.03)
≥ 2	31,677 (1.59)	0.87 (0.66–1.15)						
Unknown fever			Unknown fever			Unknown fever		
0	1,936,434 (97.01)	Reference	0	1,972,916 (98.69)	Reference	0	1,961,069 (98.24)	Reference
≥ 1	59,740 (2.99)	0.97 (0.81–1.16)	≥ 1	26,149 (1.31)	1.04 (0.83–1.29)	≥ 1	35,105 (1.76)	0.98 (0.78–1.24)
Enteric infections			Enteric infections			Enteric infections		
0	1,894,418 (94.90)	Reference	0	1,950,202 (97.56)	Reference	0	1,939,929 (97.18)	Reference
≥ 1	101,756 (5.10)	1.01 (0.88–1.16)	≥ 1	48,863 (2.44)	1.09 (0.93–1.27)	≥1	56,245 (2.82)	0.98 (0.81–1.18)
Ear infections			Ear infections			Ear infections		
0	1,926,967 (96.53)	Reference	0	1,972,285 (98.66)	Reference	0	1,951,074 (97.74)	Reference
≥ 1	69,207 (3.47)	1.15 (0.98–1.35)	≥ 1	26,780 (1.34)	0.96 (0.77–1.20)	**≥ 1**	**45**,**100 (2.26)**	**1.24 (1.03–1.49)**
Eye infections			Eye infections			Eye infections		
0	1,990,842 (99.73)	Reference	0	1,997,067 (99.90)	Reference	0	1,992,819 (99.83)	Reference
≥ 1	5,332 (0.27)	0.85 (0.46–1.58)	≥ 1	1,998 (0.10)	1.57 (0.85–2.93)	≥ 1	3,355 (0.17)	0.40 (0.13–1.24)
Urinary tract infections			Urinary tract infections			Urinary tract infections		
0	1,944,092 (97.39)	Reference	0	1,961,020 (98.10)	Reference	0	1,981,106 (99.25)	Reference
≥ 1	52,082 (2.61)	1.12 (0.93–1.34)	≥ 1	38,045 (1.90)	1.16 (0.98–1.38)	≥ 1	15,068 (0.75)	0.97 (0.68–1.36)
Skin infections			Skin infections			Skin infections		
0	1,981,528 (99.27)	Reference	0	1,989,452 (99.52)	Reference	0	1,986,639 (99.52)	Reference
≥ 1	14,646 (0.73)	1.28 (0.94–1.74)	**≥ 1**	**9**,**613 (0.48)**	**3.20 (2.61–3.92)**	≥ 1	9,535 (0.48)	1.23 (0.83–1.81)
Mycoses			Mycoses			Mycoses		
0	1,993,284 (99.86)	Reference	0	1,997,059 (99.90)	Reference	0	1,995,274 (99.95)	Reference
** ≥ 1**	**2**,**890 (0.14)**	**1.88 (1.06–3.32)**	≥ 1	2,006 (0.10)	1.70 (0.94–3.07)	≥ 1	900 (0.05)	1.93 (0.73–5.16)
Herpes			Herpes			Herpes		
0	1,986,955 (99.54)	Reference	0	1,995,571 (99.83)	Reference	0	1,990,329 (99.71)	Reference
≥ 1	9,219 (0.46)	1.36 (0.93–1.98)	≥ 1	3,494 (0.17)	1.05 (0.60–1.86)	≥ 1	5,845 (0.29)	1.50 (0.96–2.33)
Chickenpox			Chickenpox			Chickenpox		
0	1,993,706 (99.88)	Reference	0	1,997,965 (99.94)	Reference	0	1,994,790 (99.93)	Reference
≥ 1	2,468 (0.12)	1.24 (0.59–2.60)	≥ 1	1,100 (0.06)	1.70 (0.76–3.80)	≥ 1	1,384 (0.07)	1.21 (0.45–3.23)
Other viral infections			Other viral infections			Other viral infections		
0	1,954,401 (97.91)	Reference	0	1,977,333 (98.91)	Reference	0	1,974,941 (98.94)	Reference
≥ 1	41,773 (2.09)	1.02 (0.83–1.27)	≥ 1	21,732 (1.09)	0.98 (0.76–1.25)	≥ 1	21,233 (1.06)	1.19 (0.91–1.56)
Other bacterial infections			Other bacterial infections			Other bacterial infections		
0	1,987,205 (99.55)	Reference	0	1,995,424 (99.82)	Reference	0	1,990,763 (99.73)	Reference
≥ 1	8,969 (0.45)	1.10 (0.72–1.67)	≥ 1	3,641 (0.18)	1.38 (0.84–2.25)	≥ 1	5,411 (0.27)	0.80 (0.43–1.49)
GTIs			GTIs			GTIs		
0	1,990,070 (99.69)	Reference	0	1,992,920 (99.69)	Reference	0	1,990,070 (99.69)	Reference
≥ 1	6,104 (0.31)	1.21 (0.74–1.98)	**≥ 1**	**6**,**145 (0.31)**	**2.71 (2.06–3.57)**	≥ 1	6,104 (0.31)	1.21 (0.74–1.98)
Sex			Sex			Sex		
Male	1,020,895 (51.14)	Reference	Male	1,022,558 (51.15)	Reference	Male	1,020,895 (51.14)	Reference
** Female**	**975**,**279 (48.86)**	**1.06 (1.00–1.13)**	Female	976,507 (48.85)	0.98 (0.93–1.03)	**Female**	**975**,**279 (48.86)**	**1.06 (1.00–1.12)**
Residence			Residence			Residence		
Metropolitans	1,092,913 (54.75)	Reference	Metropolitans	1,094,387 (54.74)	Reference	Metropolitans	1,092,913 (54.75)	Reference
** Others**	**870**,**302 (43.60)**	**1.12 (1.05–1.19)**	**Others**	**871**,**658 (43.60)**	**1.13 (1.07–1.19)**	**Others**	**870**,**302 (43.60)**	**1.12 (1.05–1.19)**
Unknown	32,959 (1.65)	1.18 (0.94–1.47)	**Unknown**	**33**,**020 (1.65)**	**1.20 (1.00–1.45)**	Unknown	32,959 (1.65)	1.17 (0.94–1.47)
Income (percentile)			Income (percentile)			Income (percentile)		
17th − 20th	295,168 (14.79)	Reference	17th − 20th	295,550 (14.78)	Reference	17th − 20th	295,168 (14.79)	Reference
14th − 16th	420,121 (21.05)	0.94 (0.85–1.04)	14th − 16th	420,725 (21.05)	1.00 (0.91–1.09)	14th − 16th	420,121 (21.05)	0.94 (0.85–1.04)
11th − 13th	452,242 (22.66)	1.04 (0.94–1.14)	11th − 13th	452,889 (22.66)	1.04 (0.95–1.13)	11th − 13th	452,242 (22.66)	1.04 (0.94–1.14)
8th − 10th	367,586 (18.41)	0.93 (0.83–1.03)	8th − 10th	368,124 (18.41)	0.98 (0.89–1.07)	8th − 10th	367,586 (18.41)	0.93 (0.83–1.03)
0–7th	417,207 (20.90)	1.03 (0.93–1.14)	0–7th	417,878 (20.90)	1.06 (0.97–1.15)	0–7th	417,207 (20.90)	1.03 (0.93–1.14)
Unknown	43,850 (2.20)	1.05 (0.85–1.30)	Unknown	43,899 (2.20)	0.97 (0.80–1.16)	Unknown	43,850 (2.20)	1.05 (0.85–1.30)
Birth year			Birth year			Birth year		
2002	431,264 (21.60)	Reference	2002	431,989 (21.61)	Reference	2002	431,264 (21.60)	Reference
2003	426,789 (21.38)	0.98 (0.90–1.07)	2003	427,419 (21.38)	0.95 (0.88–1.02)	2003	426,789 (21.38)	0.98 (0.90–1.07)
2004	407,730 (20.43)	1.07 (0.97–1.17)	2004	408,321 (20.43)	0.99 (0.92–1.07)	2004	407,730 (20.43)	1.07 (0.98–1.17)
2005	372,142 (18.64)	0.99 (0.89–1.09)	2005	372,650 (18.64)	0.91 (0.84–0.99)	2005	372,142 (18.64)	0.99 (0.89–1.09)
2006	358,249 (17.95)	0.98 (0.88–1.09)	2006	358,686 (17.94)	0.88 (0.81–0.97)	2006	358,249 (17.95)	0.98 (0.88–1.10)

Statistically significant (*p* < 0.05) hazard ratios are indicated in bold

*CI* confidence interval, *URTI* upper respiratory tract infection, *LRTI* lower respiratory tract infection, *GTI* gastrointestinal tract infection

### Cancer risk for various infectious diseases based on outpatient visit frequency

Cancer risk analysis by outpatient-visit frequency largely mirrored the combined outpatient and hospitalization models (Supplementary Table S4). Over the ages of 0–4 years, higher outpatient visits were significantly associated with increased cancer risk for urinary tract infections (HR: 1.17, 95% CI: 1.07–1.27), one skin infection episode (HR: 1.07, 95% CI: 1.00–1.15), ≥ 2 skin infection episodes (HR: 1.10, 95% CI: 1.02–1.20), ≥ 2 mycoses episodes (HR: 1.23, 95% CI: 1.07–1.42), and ≥ 1 episode of other bacterial infections (HR: 1.16, 95% CI: 1.01–1.33). During age 0–1 year, only ≥ 1 urinary tract infection episode (HR: 1.22, 95% CI: 1.09–1.35) and ≥ 2 mycoses episodes (HR: 1.17, 95% CI: 1.01–1.36) remained significant when outpatient visits were considered alone. Similarly, in the ages of 2–4 years, outpatient visits for ≥ 1 urinary tract infection episode (HR: 1.13, 95% CI: 1.02–1.26), one skin infection episode (HR: 1.09, 95% CI: 1.02–1.16), and ≥ 2 skin infection episodes (HR: 1.14, 95% CI: 1.04–1.24), were the only infections linked to a higher cancer risk. These findings are consistent with those from the combined-frequency analysis.

### Cancer risk based on overall infectious disease frequency

**Fig. 4:**
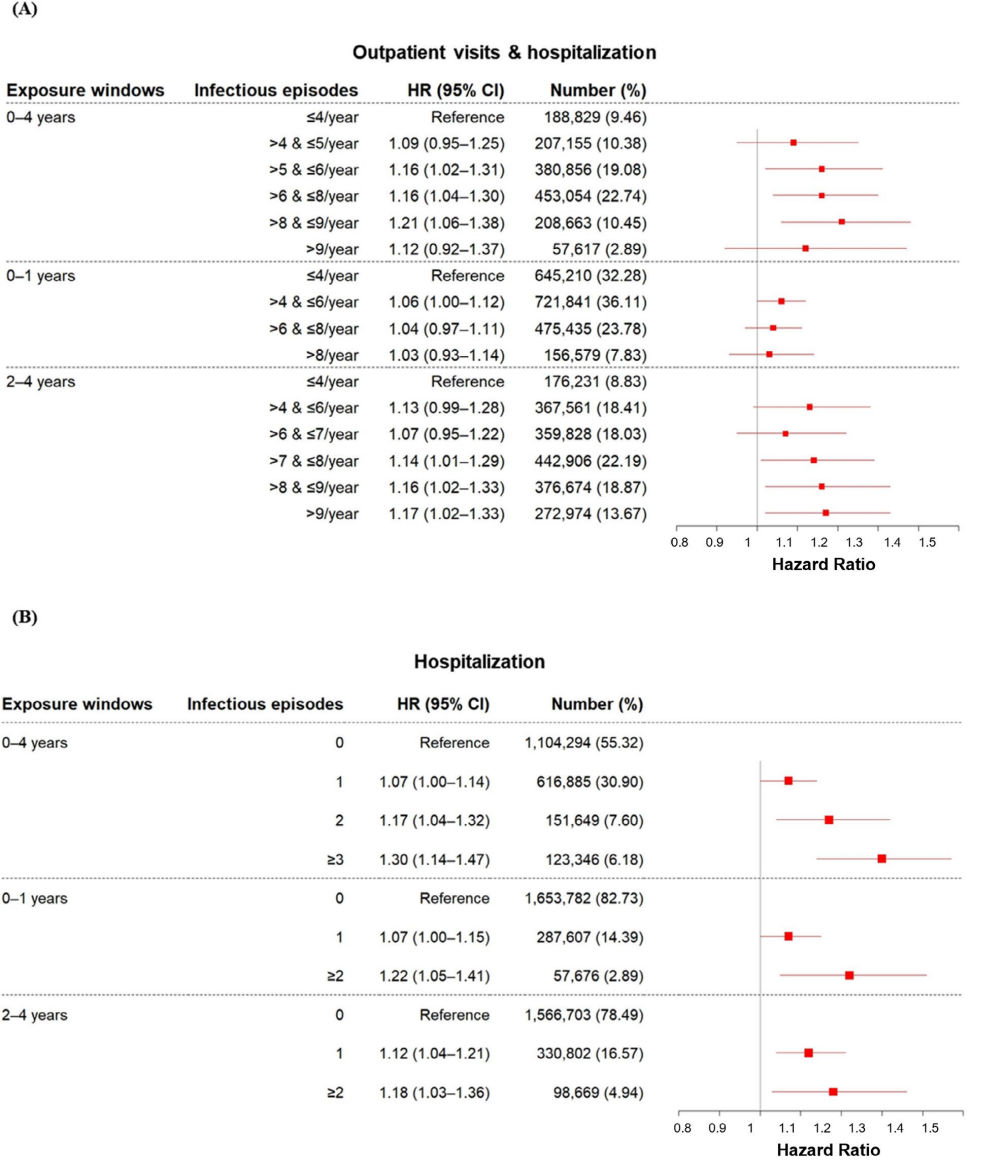
Hazard ratios for cancer incidence according to the frequency of hospital visits (**a**; combined outpatient visits and hospitalizations, **b**; hospitalizations only) for any infectious disease, during three exposure windows (ages 0–4, 0–1, and 2–4 years). Analyzes were adjusted for sex, residence, income, and birth year. Abbreviation: CI, confidence interval

Across the ages of 0–4 years, higher combined frequencies of outpatient visits and hospitalizations (≤ 6, ≤ 8, and ≤ 9 visits/year) were associated with elevated cancer risk (HRs: 1.16–1.21). An examination of hospitalization alone revealed that risk rose in a dose-response manner: one admission (HR: 1.07, 95% CI: 1.00–1.14), two admissions (HR: 1.17, 95% CI: 1.04–1.32), and ≥ 3 admissions (HR: 1.30, 95% CI: 1.14–1.47; Fig. 4). In children aged 0–1 year, only those with > 4–≤6 combined visits/year showed a modest risk increase (HR: 1.06, 95% CI: 1.00–1.12), whereas hospitalization alone was significant for one episode (HR: 1.07, 95% CI: 1.00–1.15) and > 2 episodes (HR: 1.22, 95% CI: 1.05–1.41). For ages 2–4 years, combined frequencies of ≤ 8, ≤9, and > 9 visits/year conferred a higher risk (HRs: 1.14–1.17), and hospitalization for one (HR: 1.12, 95% CI: 1.04–1.21) or > 2 episodes (HR: 1.18, 95% CI: 1.03–1.36) remained significant. Alone, outpatient visits were only intermittently significant (supplementary table S5). Overall, compared with combined or outpatient visit frequencies, hospitalization frequency demonstrated a stronger and more consistent dose-response relationship with childhood cancer risk (Fig. 4).

### Analysis of each type of cancer risk based on overall infectious disease frequency

We analyzed the association between the frequency of infectious disease-related hospital utilization and cancer risk within each cancer type’s subgroups (Supplementary Tables S6–S7). For combined outpatient visits and hospitalizations in the 0–4-year exposure window, soft tissue sarcoma showed the greatest risk among children with ≥ 9 visits/year (HR: 3.01, 95% CI: 1.41–6.43). All other cancers rose progressively: HR: 1.25, 95% CI: 1.01–1.55 at 5–6 visits; HR: 1.29, 95% CI: 1.06–1.57 at 6–8 visits; and HR: 1.41, 95% CI: 1.12–1.79 at 8–9 visits. In the 0–1-year window, soft tissue sarcoma remained elevated at > 8 visits (HR: 1.51, 95% CI: 1.02–2.25), Hodgkin lymphoma risk was reduced at 4–6 visits (HR: 0.53, 95% CI: 0.30–0.95), and all other cancers had a HR of 1.14, 95% CI: 1.03–1.27 at 4–6 visits. Between the ages of 2–4 years, lymphoid/hematopoietic cancers increased at 4–6 visits (HR: 1.23, 95% CI: 1.00–1.53), Hodgkin lymphoma decreased at 4–6 and ≥ 9 visits (HR: 0.28, 95% CI: 0.11–0.77 and HR: 0.28, 95% CI: 0.10–0.80, respectively), and soft tissue sarcoma peaked at 8–9 visits (HR: 2.39, 95% CI: 1.25–4.58).

Considering hospitalization alone, lymphoid/hematopoietic cancers rose in the 0–4-year window among children with > 3 admissions (HR: 1.47, 95% CI: 1.21–1.78), with lymphoid leukemia increasing at two admissions (HR: 1.35, 95% CI: 1.00–1.81), non-Hodgkin lymphoma at one admission (HR: 1.24, 95% CI: 1.01–1.53), central nervous system cancers at one (HR: 1.20, 95% CI: 1.03–1.41) and > 3 admissions (HR: 1.41, 95% CI: 1.05–1.88), and brain tumors at one (HR: 1.21, 95% CI: 1.03–1.42) and > 3 admissions (HR: 1.36, 95% CI: 1.01–1.83). The 0–1-year hospitalization analysis revealed that combined, all lymphomas rose at > 2 admissions (HR: 1.62, 95% CI: 1.09–2.40), with non-Hodgkin lymphoma rising at > 2 admissions (HR: 1.54, 95% CI: 1.02–2.34), while other cancers rose at > 2 admissions (HR: 1.28, 95% CI: 1.00–1.65). In the 2–4-year hospitalization window, lymphoid/hematopoietic cancers increased at one admission (HR: 1.15, 95% CI: 1.01–1.31), and other cancers at > 2 admissions (HR: 1.32, 95% CI: 1.04–1.67).

Combined visits showed inverse trends for lymphoid leukemia and renal cancer in the 0–1-year window, and for Hodgkin lymphoma across all windows, each trending toward a lower incidence with increasing infections, although not statistically significantly. However, in the 0–1-year window, when hospitalizations were considered alone, this protective pattern disappeared for all cancers, except renal cancer, with most cancers exhibiting significant or upward risk trends with increasing hospitalization frequency.

## Discussion

This nationwide cohort study provides compelling evidence that early-life infections of unusual severity or frequency are linked to increased childhood cancer risk. Urinary tract infections, severe skin infections, systemic fungal infections, and infections requiring hospitalization were associated with significantly higher cancer incidence. Moreover, the need for hospitalization and a greater number of infection episodes demonstrated a clear dose-response relationship with cancer risk. Notably, cancer rates were highest in children with multiple hospitalizations for infections, suggesting that cumulative severe infections are particularly impactful.

Compared with previous small-scale case–control studies, this large, nationwide, population-based study involves nearly two million children and medically verified registry data [8, 9, 17–19], which enabled infection classification by type, timing, care setting (hospitalization or outpatient), and frequency. We also segmented exposure into biologically and socially-relevant intervals (0–24 months and 24–60 months), considering contemporary childcare patterns, such as increased daycare attendance after the age of two years [27] and ongoing immune “training” beyond the first year after birth [28–31]. This approach allowed a more precise assessment of how childhood cancer risk is influenced by infection exposure during key postnatal immune development windows. This study’s strengths include minimized recall bias because of the use of recorded diagnoses rather than parent-reported infections and the ability to evaluate dose-response relationships across the full infection severity spectrum. However, it is limited by not capturing mild infections that are managed at home, possibly leading to an underestimation of any protective effect of these common infections against cancer [14, 15]. Additionally, despite adjustment for key confounders, residual confounding from unmeasured factors like subclinical immune defects or environmental exposures cannot be ruled out. Moreover, data on other potential confounders, such as maternal infections or breastfeeding conditions, were lacking, which might influence the infection-cancer relationship.

Before 2020, most research supported the Greaves’ delayed-infection hypothesis that limited microbial exposure in infancy impairs immune maturation and raises leukemia risk [8–10, 14, 15]. However, these studies have several key limitations: 1) most were small-scale case–control studies [8, 9], 2) infection histories were solely based on maternal questionnaires, introducing recall bias [8], 3) the immunologically critical exposure window was not clearly defined [9], 4) infections were inferred via proxies like birth month, season, and birth order and were not directly measured [9], 5) infection exposures were not confined to the early-life period [10], and 6) only select pathogens and cancer types were evaluated [8–12, 19, 24]. These limitations complicate the interpretation of previous findings, leaving the question of whether early-life infections have protective or harmful effects on childhood cancer risk unresolved.

The idea that early infections are protective has been challenged by emerging studies [17–19, 22]. A 2023 Danish analysis linked higher postnatal infection frequency and viral, gastrointestinal, and urinary tract infections to increased childhood cancer risk [17], and a Canadian meta-analysis found a 2.4-fold rise in acute lymphoblastic leukemia (ALL) risk among laboratory-confirmed infections [22]. Another Canadian study reported higher ALL odds with > 2 infections per year, notably between 12 and 18 months [19]. Similarly, a Mexico City study associated first-year upper respiratory infections after birth with greater acute leukemia risk [18]. However, these predominantly small case–control studies [17, 19] did not provide dose-response data between infection frequency and cancer risk [17, 19], and involved few cancer subtypes [18, 19, 22], making it difficult to definitively establish a causal link between infection and cancer incidence. In contrast, our large cohort study comprehensively evaluated all infection types, cancer types, and the full infection severity and frequency spectrum and demonstrated a clear dose-response effect based on infection severity. Importantly, we found no evidence that mild infections reduce cancer risk. The trend was towards a higher risk with increasing infections severe enough to require medical attention.

We observed heterogeneous infection–cancer associations across cancer subtypes. Although most cancers showed positive associations, Hodgkin lymphoma exhibited an inverse relationship, with higher infection frequencies in early childhood corresponding to lower incidence, albeit based on small numbers. This is consistent with hypotheses suggesting that early microbial exposure protects against cancer, although the trend disappeared when restricted to severe infections. In contrast, soft-tissue sarcomas showed strong positive associations with infection burden, but these findings should be interpreted with caution because of the limited number of cases. Nevertheless, these subtype-specific variations highlight intriguing questions about whether some malignancies are particularly sensitive to infection-driven immune perturbations or if distinct oncogenic events are preferentially influenced by specific infections.

Clinically, our findings confirm that long-term cancer risk is not increased by routine childhood infections. However, unusually severe or recurrent infant infections warrant vigilance. Severe pediatric infections may contribute to oncogenesis through chronic inflammation-induced DNA damage, excessive proinflammatory cytokine release, compensatory cellular proliferation (expanding premalignant clones), and tumor surveillance-impairing immune exhaustion [20, 21, 32, 33]. In addition, severe infections are often treated with antibiotics, which may disrupt the developing gut microbiome. Because early-life microbiome composition plays an important role in immune system maturation, antibiotic-associated microbiome alterations could potentially influence susceptibility to immune-mediated diseases, including cancer [17]. Moreover, such infections may unmask underlying immune vulnerabilities, e.g., primary immunodeficiencies or congenital dysregulation [34], as exemplified by invasive fungal infections [35, 36] and serious bacterial infections (e.g., neonatal UTIs, deep skin infections) being rare [37, 38] in otherwise immunocompetent children. Looking ahead, key questions include the molecular and cellular pathways by which severe infections promote oncogenesis and whether stratified strategies (enhanced monitoring or prophylaxis) can reduce cancer risk in high-risk children.

Our study has several limitations. Using claims data may fail to capture mild infections, misclassify exposures or outcomes, and we used hospitalization to approximate severity. Residual confounding from household, environmental, and genetic factors likely persist despite adjustment. Although the design is longitudinal, reverse causation cannot be fully excluded if preclinical malignancy increased susceptibility to infections or healthcare contact before diagnosis.

Because this study simultaneously evaluated multiple infectious disease categories across different exposure windows, defining a single exposed versus unexposed group for baseline comparisons was not appropriate. Therefore, baseline characteristics were summarized descriptively, and the associations between infection exposures and childhood cancer risk were assessed using multivariable regression models.

One important consideration in our study design is that follow-up began at age five, after the exposure window covering the first five years of life. This approach allowed us to clearly separate the exposure assessment period from the outcome observation period and to reduce potential reverse causation, as infections occurring shortly before cancer diagnosis may represent early manifestations of undiagnosed malignancy. However, this design also means that cancer cases diagnosed before age five were not included in the analysis. As a result, our findings primarily reflect associations between early-life infections and cancer risk occurring after early childhood. Future studies incorporating earlier follow-up periods may help further clarify potential associations with cancers diagnosed at younger ages.

Another limitation of this study is the lack of information on vaccination status in the claims database. Because vaccination can influence infection susceptibility and immune system development in early life, the absence of vaccination data may represent a source of residual confounding.

In conclusion, this nationwide cohort study provides clear evidence that routine early-life infections do not increase childhood cancer risk, and that severe or recurrent infections, especially hospitalization-requiring ones, are associated with cancer risk elevation in a dose-response manner. By moving beyond the simplistic protective hygiene hypothesis, these findings clarify the complex role of infections in pediatric cancer etiology and highlight the clinical importance of monitoring children with unusually severe or frequent infections.

## Supplementary Information

Supplementary Material 1.

## Data Availability

The data underlying this study are owned by the Korea National Health Insurance Service (NHIS) and are available to qualified researchers upon approval from NHIS. Data can be requested via the NHIS data access portal (https://nhiss.nhis.or.kr). Legal and ethical restrictions prevent public sharing of individual-level data. The authors did not have any special access privileges that others would not have.

## Abbreviations

ALL: Acute lymphoblastic leukemia
CI: Confidence interval
CNS: Central nervous system
GTI: Gastrointestinal tract infection
GVIF: Generalized variance inflation factor
HR: Hazard ratio
ICD-10: International Classification of Diseases, 10th Revision
LRTI: Lower respiratory tract infection
NHIS: National Health Insurance Service (Korea)
PH: Cox proportional hazards model
STROBE: Strengthening the Reporting of Observational Studies in Epidemiology
URTI: Upper respiratory tract infection
UTI: Urinary tract infection

## References

[1] Huang J, Chan SC, Ngai CH, Lok V, Zhang L, Lucero-Prisno DE III, Xu W, Zheng ZJ, Elcarte E, Withers M. Global incidence, mortality and temporal trends of cancer in children: A joinpoint regression analysis. Cancer Med. 2023;12(2):1903–11. 35822443 10.1002/cam4.5009 PMC9883415

[2] Wu Y, Deng Y, Wei B, Xiang D, Hu J, Zhao P, Lin S, Zheng Y, Yao J, Zhai Z. Global, regional, and national childhood cancer burden, 1990–2019: An analysis based on the Global Burden of Disease Study 2019. J Adv Res. 2022;40:233–47. 35700919 10.1016/j.jare.2022.06.001 PMC9481947

[3] Sultan I, Alfaar AS, Sultan Y, Salman Z, Qaddoumi I. Trends in childhood cancer: Incidence and survival analysis over 45 years of SEER data. PLoS ONE. 2025;20(1):e0314592. 39752445 10.1371/journal.pone.0314592 PMC11698462

[4] Bhakta N, Force LM, Allemani C, Atun R, Bray F, Coleman MP, Steliarova-Foucher E, Frazier AL, Robison LL, Rodriguez-Galindo C. Childhood cancer burden: a review of global estimates. Lancet Oncol. 2019;20(1):e42–53. 30614477 10.1016/S1470-2045(18)30761-7

[5] Buser JM, Lake K, Ginier E. Environmental risk factors for childhood cancer in an era of global climate change: a scoping review. J Pediatr Health Care. 2022;36(1):46–56. 34134914 10.1016/j.pedhc.2021.05.005

[6] Navarrete-Meneses MP, Salas-Labadía C, Gómez-Chávez F, Pérez-Vera P. Environmental pollution and risk of childhood cancer: a scoping review of evidence from the last decade. Int J Mol Sci. 2024;25(6):3284. 38542255 10.3390/ijms25063284 PMC10970446

[7] Hoang TT, Scheurer ME, Lupo PJ. Overview of the etiology of childhood cancer and future directions. Curr Opin Pediatr. 2025;37(1):59–66. 39699102 10.1097/MOP.0000000000001419

[8] Urayama KY, Ma X, Selvin S, Metayer C, Chokkalingam AP, Wiemels JL, Does M, Chang J, Wong A, Trachtenberg E. Early life exposure to infections and risk of childhood acute lymphoblastic leukemia. Int J Cancer. 2011;128(7):1632–43. 21280034 10.1002/ijc.25752 PMC3165002

[9] Marcotte EL, Ritz B, Cockburn M, Yu F, Heck JE. Exposure to infections and risk of leukemia in young children. Cancer Epidemiol Biomarkers Prev. 2014;23(7):1195–203. 24793957 10.1158/1055-9965.EPI-13-1330 PMC4100471

[10] Lin J-N, Lin C-L, Lin M-C, Lai C-H, Lin H-H, Yang C-H, Sung F-C, Kao C-H. Risk of leukaemia in children infected with enterovirus: a nationwide, retrospective, population-based, Taiwanese-registry, cohort study. Lancet Oncol. 2015;16(13):1335–43. 26321214 10.1016/S1470-2045(15)00060-1

[11] Rudant J, Lightfoot T, Urayama KY, Petridou E, Dockerty JD, Magnani C, Milne E, Spector LG, Ashton LJ, Dessypris N. Childhood acute lymphoblastic leukemia and indicators of early immune stimulation: a Childhood Leukemia International Consortium study. Am J Epidemiol. 2015;181(8):549–62. 25731888 10.1093/aje/kwu298 PMC4850899

[12] Rudant J, Orsi L, Menegaux F, Petit A, Baruchel A, Bertrand Y, Lambilliotte A, Robert A, Michel G, Margueritte G. Childhood acute leukemia, early common infections, and allergy: The ESCALE Study. Am J Epidemiol. 2010;172(9):1015–27. 20807738 10.1093/aje/kwq233

[13] Kinlen L. Evidence for an infective cause of childhood leukaemia: comparison of a Scottish new town with nuclear reprocessing sites in Britain. Lancet. 1988;332(8624):1323–7. 10.1016/s0140-6736(88)90867-7 2904050

[14] Greaves M. Infection, immune responses and the aetiology of childhood leukaemia. Nat Rev Cancer. 2006;6(3):193–203. 16467884 10.1038/nrc1816

[15] Greaves M. A causal mechanism for childhood acute lymphoblastic leukaemia. Nat Rev Cancer. 2018;18(8):471–84. 29784935 10.1038/s41568-018-0015-6 PMC6986894

[16] Hauer J, Fischer U, Borkhardt A. Toward prevention of childhood ALL by early-life immune training. Blood J Am Soc Hematol. 2021;138(16):1412–28. 10.1182/blood.2020009895 PMC8532195 34010407

[17] Sirirungreung A, Hansen J, Ritz B, Heck JE. Association between medically diagnosed postnatal infection and childhood cancers: a matched case-control study in Denmark, 1978 to 2016. Int J Cancer. 2023;153(5):994–1002. 37243370 10.1002/ijc.34604 PMC10524667

[18] Sepúlveda-Robles O, Flores-Lujano J, Núñez-Enríquez JC, Jiménez-Hernández E, Duarte-Rodríguez DA, Martín-Trejo JA, Espinoza-Hernández LE, García-Jiménez X, Paredes-Aguilera R, Dosta-Herrera JJ. Early Infection Incidence and Risk of Acute Leukemia Development Among Mexican Children. Cancers. 2025;17(5):733. 40075581 10.3390/cancers17050733 PMC11899646

[19] Hwee J, Sutradhar R, Kwong JC, Sung L, Cheng S, Pole JD. Infections and the development of childhood acute lymphoblastic leukemia: a population-based study. Eur J Cancer Prev. 2020;29(6):538–45. 32032155 10.1097/CEJ.0000000000000564

[20] Blank CU, Haining WN, Held W, Hogan PG, Kallies A, Lugli E, Lynn RC, Philip M, Rao A, Restifo NP, et al. Defining ‘T cell exhaustion’. Nat Rev Immunol. 2019;19(11):665–74. 31570879 10.1038/s41577-019-0221-9 PMC7286441

[21] Mella C, Tsarouhas P, Brockwell M, Ball HC. The Role of Chronic Inflammation in Pediatric Cancer. Cancers (Basel). 2025;17(1):154. 10.3390/cancers17010154 PMC11719864 39796780

[22] Hwee J, Tait C, Sung L, Kwong JC, Sutradhar R, Pole JD. A systematic review and meta-analysis of the association between childhood infections and the risk of childhood acute lymphoblastic leukaemia. Br J Cancer. 2018;118(1):127–37. 29065105 10.1038/bjc.2017.360 PMC5765221

[23] Jacqueline C, Tasiemski A, Sorci G, Ujvari B, Maachi F, Missé D, Renaud F, Ewald P, Thomas F, Roche B. Infections and cancer: the fifty shades of immunity hypothesis. BMC Cancer. 2017;17(1):257. 28403812 10.1186/s12885-017-3234-4 PMC5389015

[24] Gilham C, Peto J, Simpson J, Roman E, Eden TO, Greaves MF, Alexander FE. Day care in infancy and risk of childhood acute lymphoblastic leukaemia: findings from UK case-control study. BMJ. 2005;330(7503):1294. 15849205 10.1136/bmj.38428.521042.8F PMC558199

[25] Cheol Seong S, Kim Y-Y, Khang Y-H, Heon Park J, Kang H-J, Lee H, Do C-H, Song J-S, Hyon Bang J, Ha S. Data resource profile: the national health information database of the National Health Insurance Service in South Korea. Int J Epidemiol. 2017;46(3):799–800. 27794523 10.1093/ije/dyw253 PMC5837262

[26] Kim MK, Han K, Lee S-H. Current trends of big data research using the Korean National Health Information Database. Diabetes metabolism J. 2022;46(4):552–63. 10.4093/dmj.2022.0193 PMC9353560 35929173

[27] OECD, Family Database. PF3.2: Enrolment in childcare and pre-school. 2024.

[28] Yatsunenko T, Rey FE, Manary MJ, Trehan I, Dominguez-Bello MG, Contreras M, Magris M, Hidalgo G, Baldassano RN, Anokhin AP. Human gut microbiome viewed across age and geography. nature. 2012;486(7402):222–227. 10.1038/nature11053 PMC3376388 22699611

[29] Ygberg S, Nilsson A. The developing immune system–from foetus to toddler. Acta Paediatr. 2012;101(2):120–7. 22003882 10.1111/j.1651-2227.2011.02494.x

[30] Henneke P, Kierdorf K, Hall LJ, Sperandio M, Hornef M. Perinatal development of innate immune topology. Elife. 2021;10:e67793. 34032570 10.7554/eLife.67793 PMC8149122

[31] Kumar BV, Connors TJ, Farber DL. Human T Cell Development, Localization, and Function throughout Life. Immunity. 2018;48(2):202–13. 29466753 10.1016/j.immuni.2018.01.007 PMC5826622

[32] Zhao H, Wu L, Yan G, Chen Y, Zhou M, Wu Y, Li Y. Inflammation and tumor progression: signaling pathways and targeted intervention. Signal Transduct Target therapy. 2021;6(1):263. 10.1038/s41392-021-00658-5 PMC8273155 34248142

[33] Tripathi S, Sharma Y, Kumar D. Unveiling the link between chronic inflammation and cancer. Metabol Open. 2025;25:100347. 39876904 10.1016/j.metop.2025.100347 PMC11772974

[34] Kebudi R, Kiykim A, Sahin MK. Primary immunodeficiency and cancer in children; a review of the literature. Curr Pediatr reviews. 2019;15(4):245–50. 10.2174/1573396315666190917154058 PMC7040504 31530267

[35] Calle-Miguel L, Garrido-Colino C, Santiago-García B, Moreno Santos MP, Gonzalo Pascual H, Ponce Salas B, Beléndez Bieler C, Navarro Gómez M, Guinea Ortega J. Rincón-López EM: Changes in the epidemiology of invasive fungal disease in a Pediatric Hematology and Oncology Unit: the relevance of breakthrough infections. BMC Infect Dis. 2023;23(1):348. 37226103 10.1186/s12879-023-08314-9 PMC10210274

[36] Lionakis MS. Primary immunodeficiencies and invasive fungal infection: when to suspect and how to diagnose and manage. Curr Opin Infect Dis. 2019;32(6):531–7. 31567735 10.1097/QCO.0000000000000593

[37] Godaly G, Ambite I, Svanborg C. Innate immunity and genetic determinants of urinary tract infection susceptibility. Curr Opin Infect Dis. 2015;28(1):88–96. 25539411 10.1097/QCO.0000000000000127 PMC4286230

[38] de Wit J, Brada RJ, van Veldhuizen J, Dalm VA, Pasmans SG. Skin disorders are prominent features in primary immunodeficiency diseases: A systematic overview of current data. Allergy. 2019;74(3):464–82. 30480813 10.1111/all.13681

