# Supplementary material for: Early-life infections and childhood cancer risk: a nationwide cohort study of 2 million children
Source: BMC Cancer. 2026 Apr 13;26:645. doi: 10.1186/s12885-026-15927-1 (PMC13188238; doi:10.1186/s12885-026-15927-1)
Supplement: Supplementary file 1 — Supplementary Material 1. [file 12885_2026_15927_MOESM1_ESM.docx]

**Early-life infections and childhood cancer risk: a nationwide cohort study of 2 million children**

Supplementary materials

**Supplementary Table S1.** Infectious diseases and ICD-10 codes.

**Supplementary Table S2.** Cancer types and ICD-10 codes.

**Supplementary Table S3.** The baseline characteristics of Cohorts 1 and 2.

**Supplementary Table S4.** Hazard ratios for cancer incidence based on hospital visit frequency (outpatient visits only) for each infectious disease type during three exposure windows (ages 0–4, 0–1, and 2–4 years).

**Supplementary Table S5.** Hazard ratios for cancer incidence based on hospital visit frequency (outpatient visits only) for any infectious disease during three exposure windows (ages 0–4, 0–1, and 2–4 years).

**Supplementary Table S6.** Hazard ratios for each cancer subtype incidence based on hospital visit frequency (outpatient visits or hospitalizations) for any infectious disease during three exposure windows (ages 0–4, 0–1, and 2–4 years).

**Supplementary Table S7.** Hazard ratios for each cancer subtype incidence based on hospital visit frequency (hospitalizations only) for any infectious disease during the three exposure windows (ages 0–4, 0–1, and 2–4 years).

**Table S1.** Infectious diseases and ICD-10 codes

| Infectious diseases | ICD-10 codes |
| --- | --- |
| URTIs | J00-J06 |
| Influenza | J09, J10, J11 |
| Pneumonia | J12, J13, J14, J15, J16, J17, J18 |
| LRTIs | J20, J21, J22 |
| Unknown fever | R50 |
| Enteric infections | A00-A08 |
| Ear infections | H600, H601, H602, H603, H608, H609, H62, H65, H66, H67, H70, H730, H750, H830, H940 |
| Eye infections | H000, H03, H043, H050, H09, H100, H102, H103, H130, H131, H160, H190, H191, H192, H220, H440, H451, B30 |
| Urinary tract infections | N300, N341, N351, N37, N390 |
| Skin infections | L00-L08, A46, B86 |
| Mycoses | B35-B39, B40-B49 |
| Herpes | B00 |
| Chickenpox | B01 |
| Other viral infections | B02-B09, B25-B27, B33, B34, B97, A90-A99 |
| Other bacterial infections | A20-A29, A30-A38, A42-A44, A48, A49, A65-A69, A70-A79, B95, B96 |
| GTIs | K230, K231, K25-K28, K293, K294, K295, K35-K37, K61, K630, K632, K650, K678, K908, K930 |

ICD: international classification of diseases, URTI: upper respiratory tract infection, LRTI: lower respiratory tract infection, GTI: gastrointestinal tract infection.

**Table S2.** Cancer types and ICD-10 codes

| Cancer types | ICD-10 codes |
| --- | --- |
| Lymphoid and hematopoietic cancer | C81-C96 |
| Leukemia | C91-C95 |
| Lymphoid leukemia | C91 |
| Myeloid leukemia | C92 |
| Lymphoma | C81-C86 |
| Hodgkin lymphoma | C81 |
| Non-Hodgkin lymphoma | C82-C86 |
| CNS cancer | C69-C72 |
| Brain cancer | C70-C72 |
| Bone cancer | C40, C41 |
| Soft tissue sarcoma | C47, C49 |
| Renal cancer | C64 |
| Liver cancer | C22 |

ICD: International Classification of Diseases, CNS: central nervous system.

**Table S3.** The baseline characteristics of Cohorts 1 and 2.

|  | Cohort 1 (n = 1,996,174) | | |  |  | Cohort 2 (n = 1,999,065) | | |
| --- | --- | --- | --- | --- | --- | --- | --- | --- |
|  | No cancer  (n=1,991,898) | Cancer  (n=4,276) | p-value |  |  | No cancer  (n=1,992,956) | Cancer  (n=6,109) | p-value |
|  | n, (%) | n, (%) |  |  |  | n, (%) | n, (%) |  |
| Sex |  |  | 0.09 |  | Sex |  |  | 0.22 |
| Male | 1,018,763 (51.15) | 2,132 (49.86) |  |  | Male | 1,019,385 (51.15) | 3,173 (51.94) |  |
| Female | 973,135 (48.85) | 2,144 (50.14) |  |  | Female | 973,571 (48.85) | 2,936 (48.06) |  |
| Residence |  |  | <.0001 |  | Residence |  |  | <.0001 |
| Metropolitans | 1,090,714 (54.76) | 2,199 (51.43) |  |  | Metropolitans | 1,091,256 (54.76) | 3,131 (51.25) |  |
| Others | 868,308 (43.59) | 1,994 (46.63) |  |  | Others | 868,800 (43.59) | 2,858 (46.78) |  |
| Unknown | 32,873 (1.65) | 83 (1.94) |  |  | Unknown | 32,900 (1.65) | 120 (1.96) |  |
| Income (percentile) |  |  | 0.03 |  | Income (percentile) |  |  | 0.07 |
| 17^th^–20^th^ | 294,543 (14.79) | 625 (14.62) |  |  | 17^th^–20^th^ | 294,679 (14.79) | 871 (14.26) |  |
| 14^th^–16^th^ | 419,281 (21.05) | 840 (19.64) |  |  | 14^th^–16^th^ | 419,484 (21.05) | 1,241 (20.31) |  |
| 11^th^–13^th^ | 451,229 (22.65) | 1,013 (23.69) |  |  | 11^th^–13^th^ | 451,474 (22.65) | 1,415 (23.16) |  |
| 8^th^–10^th^ | 366,840 (18.42) | 746 (17.45) |  |  | 8^th^–10^th^ | 367,033 (18.42) | 1,091 (17.86) |  |
| 0–7^th^ | 416,256 (20.90) | 951 (22.24) |  |  | 0–7^th^ | 416,517 (20.90) | 1,361 (22.28) |  |
| Unknown | 43,749 (2.20) | 101 (2.36) |  |  | Unknown | 43,769 (2.20) | 130 (2.13) |  |
| Year of birth |  |  | <.0001 |  | Year of birth |  |  | <.0001 |
| 2002 | 430,085 (21.59) | 1,179 (27.57) |  |  | 2002 | 430,345 (21.59) | 1,644 (26.91) |  |
| 2003 | 425,802 (21.38) | 987 (23.08) |  |  | 2003 | 426,022 (21.38) | 1,397 (22.87) |  |
| 2004 | 406,834 (20.42) | 896 (20.95) |  |  | 2004 | 407,058 (20.42) | 1,263 (20.67) |  |
| 2005 | 371,479 (18.65) | 663 (15.51) |  |  | 2005 | 371,677 (18.65) | 973 (15.93) |  |
| 2006 | 357,698 (17.96) | 551 (12.89) |  |  | 2006 | 357,854 (17.96) | 832 (13.62) |  |

**Table S4.** Hazard ratios for cancer incidence based on hospital visit frequency (outpatient visits only) for each infectious disease type during three exposure windows (ages 0–4, 0–1, and 2–4 years).

| **0–4 years old** | | |  | **0–1 years old** | | |  | **2–4 years old** | | |
| --- | --- | --- | --- | --- | --- | --- | --- | --- | --- | --- |
| Infectious diseases, episodes | n (%) | Hazard ratio  (95% CI) |  | Infectious diseases, episodes | n (%) | Hazard Ratio  (95% CI) |  | Infectious diseases, episodes | n (%) | Hazard ratio  (95% CI) |
| URTIs | | |  | URTIs | | |  | URTIs | | |
| <2/year | 223,051 (11.17) | Reference |  | <2/year | 483,631 (24.19) | Reference |  | <2/year | 239,981 (12.02) | Reference |
| ≥2 & <3/year | 299,989 (15.03) | 1.09 (0.97–1.24) |  | ≥2 & <3/year | 402,157 (20.12) | 1.00 (0.93–1.08) |  | ≥2 & <4/year | 528,130 (26.46) | 1.07 (0.96–1.20) |
| ≥3 & <4/year | 416,254 (20.85) | 1.02 (0.91–1.16) |  | ≥3 & <4/year | 399,136 (19.97) | 0.99 (0.91–1.07) |  | ≥4 & <6/year | 655,047 (32.82) | 1.07 (0.96–1.19) |
| ≥4 & <5/year | 437,242 (21.90) | 1.06 (0.94–1.20) |  | ≥4 & <6/year | 523,472 (26.19) | 1.00 (0.93–1.08) |  | ≥6 & <8/year | 450,380 (22.56) | 1.10 (0.98–1.24) |
| ≥5 & <6/year | 343,233 (17.19) | 1.07 (0.95–1.22) |  | ≥6/year | 190,669 (9.54) | 1.03 (0.93–1.14) |  | ≥8/year | 122,636 (6.14) | 1.12 (0.96–1.30) |
| ≥6/year | 276,405 (13.85) | 1.10 0.97–1.26) |  |  |  |  |  |  |  |  |
| Influenza | | |  | Influenza | | |  | Influenza | | |
| 0 | 1,769,568 (88.65) | Reference |  | 0 | 1,973,932 (98.74) | Reference |  | 0 | 1,787,983 (89.57) | Reference |
| ≥1 | 226,606 (11.35) | 1.01 (0.90–1.12) |  | ≥1 | 25,133 (1.26) | 1.00 (0.80–1.24) |  | ≥1 | 208,191 (10.43) | 1.00 (0.89–1.12) |
| Pneumonia | | |  | Pneumonia | | |  | Pneumonia | | |
| 0 | 827,360 (41.45) | Reference |  | 0 | 1,440,328 (72.05) | Reference |  | 0 | 1,051,183 (52.66) | Reference |
| 1 | 530,601 (26.58) | 0.99 (0.91–1.06) |  | 1 | 368,380 (18.43) | 0.94 (0.87–1.00) |  | 1 | 468,103 (23.45) | 1.04 (0.97–1.12) |
| 2–4 | 476,373 (23.86) | 0.99 (0.92–1.08) |  | 2–3 | 151,945 (7.60) | 1.03 (0.93–1.13) |  | 2–3 | 316,368 (15.85) | 1.03 (0.94–1.12) |
| ≥5 | 161,840 (8.11) | 0.99 (0.88–1.12) |  | ≥4 | 38,412 (1.92) | 1.08 (0.90–1.30) |  | ≥4 | 160,520 (8.04) | 0.99 (0.88–1.11) |
| LRTIs | | |  | LRTIs | | |  | LRTIs | | |
| <1/year | 261,116 (13.08) | Reference |  | <2/year | 1,120,424 (56.05) | Reference |  | <2/year | 601,591 (30.14) | Reference |
| ≥1 & <2/year | 440,626 (22.07) | 1.06 (0.96–1.18) |  | ≥2 & <4/year | 627,764 (31.40) | 1.05 (0.99–1.12) |  | ≥2 & <4/year | 697,321 (34.93) | 0.95 (0.88–1.02) |
| ≥2 & <3/year | 484,357 (24.26) | 1.05 (0.94–1.17) |  | ≥4/year | 250,877 (12.55) | 0.93 (0.85–1.02) |  | ≥4 & <6/year | 480,350 (24.06) | 1.02 (0.93–1.11) |
| ≥3 & <4/year | 391,628 (19.62) | 1.09 (0.97–1.22) |  |  |  |  |  | ≥6/year | 216,912 (10.87) | 0.96 (0.85–1.07) |
| ≥4 & <5/year | 247,620 (12.40) | 1.08 (0.95–1.23) |  |  |  |  |  |  |  |  |
| ≥5/year | 170,827 (8.56) | 0.96 (0.83–1.11) |  |  |  |  |  |  |  |  |
| Unknown fever | | |  | Unknown fever | | |  | Unknown fever | | |
| 0 | 1,145,899 (57.40) | Reference |  | 0 | 1,573,279 (78.70) | Reference |  | 0 | 1,380,322 (69.15) | Reference |
| 1 | 549,585 (27.53) | 1.05 (0.98–1.13) |  | 1 | 341,198 (17.07) | 1.02 (0.96–1.09) |  | 1 | 425,869 (21.33) | 1.04 (0.97–1.12) |
| ≥2 | 300,690 (15.06) | 1.04 (0.95–1.13) |  | ≥2 | 84,588 (4.23) | 1.10 (0.97–1.25) |  | ≥2 | 189,983 (9.52) | 0.97 (0.87–1.09) |
| Enteric | | |  | Enteric infections | | |  | Enteric infections | | |
| 0 | 1,065,506 (53.38) | Reference |  | 0 | 1,469,643 (73.52) | Reference |  | 0 | 1,339,287 (67.09) | Reference |
| 1 | 529,708 (26.54) | 1.02 (0.95–1.09) |  | 1 | 374,229 (18.72) | 1.01 (0.94–1.07) |  | 1 | 424,165 (21.25) | 1.00 (0.93–1.08) |
| ≥2 | 400,960 (20.09) | 1.02 (0.94–1.11) |  | ≥2 | 155,193 (7.76) | 1.10 (1.00–1.21) |  | ≥2 | 232,722 (11.66) | 1.02 (0.92–1.13) |
| Ear infections | | |  | Ear infections | | |  | Ear infections | | |
| 0 | 361,819 (18.13) | Reference |  | 0 | 1,023,063 (51.18) | Reference |  | 0 | 553,595 (27.73) | Reference |
| 1–2 | 675,810 (33.86) | 0.93 (0.85–1.01) |  | 1–2 | 652,493 (32.64) | 0.98 (0.92–1.04) |  | 1–2 | 727,371 (36.44) | 0.96 (0.88–1.04) |
| 3–4 | 362,096 (18.14) | 0.98 (0.89–1.09) |  | 3–4 | 192,657 (9.64) | 0.98 (0.89–1.07) |  | 3–4 | 322,080 (16.13) | 0.98 (0.93–1.12) |
| ≥5 | 596,449 (29.88) | 0.95 (0.87–1.05) |  | ≥5 | 130,852 (6.55) | 1.00 (0.89–1.11) |  | ≥5 | 392,828 (19.68) | 1.06 (0.91–1.09) |
| Eye infections | | |  | Eye infections | | |  | Eye infections | | |
| 0 | 833,281 (41.74) | Reference |  | 0 | 1,485,000 (74.28) | Reference |  | 0 | 1,068,091 (53.51) | Reference |
| 1 | 692,671 (34.70) | 0.96 (0.90–1.03) |  | 1 | 404,501 (20.23) | 0.99 (0.93–1.06) |  | 1 | 616,318 (30.87) | 0.98 (0.91–1.05) |
| ≥2 | 470,222 (23.56) | 1.01 (0.93–1.09) |  | ≥2 | 109,564 (5.48) | 0.95 (0.84–1.07) |  | ≥2 | 311,765 (15.62) | 1.09 (1.00–1.19) |
| Urinary tract infections | | |  | Urinary tract infections | | |  | Urinary tract infections | | |
| 0 | 1,738,693 (87.10) | Reference |  | 0 | 1,893,083 (94.70) | Reference |  | 0 | 1,825,134 (91.43) | Reference |
| ≥1 | 257,481 (12.90) | 1.17 (1.07–1.27) |  | ≥1 | 105,982 (5.30) | 1.22 (1.09–1.35) |  | ≥1 | 171,040 (8.57) | 1.13 (1.02–1.26) |
| Skin infections | | |  | Skin infections | | |  | Skin infections | | |
| 0 | 953,431 (47.76) | Reference |  | 0 | 1,587,964 (79.44) | Reference |  | 0 | 1,157,334 (57.98) | Reference |
| 1 | 650,769 (32.60) | 1.07 (1.00–1.15) |  | 1 | 334,444 (16.73) | 0.99 (0.93–1.06) |  | 1 | 560,625 (28.08) | 1.09 (1.02–1.16) |
| ≥2 | 391,974 (19.64) | 1.10 (1.02–1.20) |  | ≥2 | 76,657 (3.83) | 0.99 (0.86–1.14) |  | ≥2 | 278,215 (13.94) | 1.14 (1.04–1.24) |
| Mycoses | | |  | Mycoses | | |  | Mycoses | | |
| 0 | 1,577,669 (79.03) | Reference |  | 0 | 1,711,464 (85.61) | Reference |  | 0 | 1,834,464 (91.90) | Reference |
| 1 | 331,824 (16.62) | 1.02 (0.94–1.10) |  | 1 | 233,926 (11.70) | 1.04 (0.96–1.12) |  | 1 | 161,710 (8.10) | 1.05 (0.94–1.17) |
| ≥2 | 86,681 (4.34) | 1.23 (1.07–1.42) |  | ≥2 | 53,675 (2.69) | 1.17 (1.01–1.36) |  | ≥2 | 1,834,464 (91.90) | Reference |
| Herpes | | |  | Herpes | | |  | Herpes | | |
| 0 | 1,673,043 (83.81) | Reference |  | 0 | 1,887,980 (94.44) | Reference |  | 0 | 1,757,037 (88.02) | Reference |
| ≥1 | 323,131 (16.19) | 1.01 (0.93–1.10) |  | ≥1 | 111,085 (5.56) | 1.00 (0.90–1.12) |  | ≥1 | 239,137 (11.98) | 1.00 (0.91–1.09) |
| Chickenpox | | |  | Chickenpox | | |  | Chickenpox | | |
| 0 | 1,681,823 (84.25) | Reference |  | 0 | 1,938,082 (96.95) | Reference |  | 0 | 1,738,316 (87.08) | Reference |
| ≥1 | 314,351 (15.75) | 1.06 (0.98–1.15) |  | ≥1 | 60,983 (3.05) | 1.14 (0.99–1.30) |  | ≥1 | 257,858 (12.92) | 1.04 (0.95–1.14) |
| Other viral infections | | |  | Other viral infections | | |  | Other viral infections | | |
| 0 | 967,482 (48.47) | Reference |  | 0 | 1,509,035 (75.49) | Reference |  | 0 | 1,225,199 (61.38) | Reference |
| 1 | 643,319 (32.23) | 0.99 (0.92–1.06) |  | 1 | 394,215 (19.72) | 1.04 (0.97–1.10) |  | 1 | 520,294 (26.06) | 1.02 (0.95–1.09) |
| ≥2 | 385,373 (19.31) | 1.00 (0.92–1.09) |  | ≥2 | 95,815 (4.79) | 0.95 (0.83–1.08) |  | ≥2 | 250,681 (12.56) | 1.00 (0.91–1.10) |
| Other bacterial infections | | |  | Other bacterial infections | | |  | Other bacterial infections | | |
| 0 | 1,911,539 (95.76) | Reference |  | 0 | 1,972,860 (98.69) | Reference |  | 0 | 1,931,052 (96.74) | Reference |
| ≥1 | 84,635 (4.24) | 1.16 (1.01–1.33) |  | ≥1 | 26,205 (1.31) | 1.17 (0.95–1.44) |  | ≥1 | 65,122 (3.26) | 1.09 (0.93–1.28) |
| Gastrointestinal tract infections | | |  | GTIs | | |  | GTIs | | |
| 0 | 1,943,551 (97.36) | Reference |  | 0 | 1,978,291 (98.96) | Reference |  | 0 | 1,962,221 (98.30) | Reference |
| ≥1 | 52,623 (2.64) | 1.11 (0.93–1.33) |  | ≥1 | 20,774 (1.04) | 0.95 (0.73–1.22) |  | ≥1 | 33,953 (1.70) | 1.07 (0.85–1.34) |
| Sex | | |  | Sex | | |  | Sex | | |
| Male | 1,020,895 (51.14) | Reference |  | Male | 1,022,558 (51.15) | Reference |  | Male | 1,020,895 (51.14) | Reference |
| Female | 975,279 (48.86) | 1.06 (1.00–1.13) |  | Female | 976,507 (48.85) | 0.97 (0.93–1.02) |  | Female | 975,279 (48.86) | 1.06 (1.00–1.13) |
| Residence | | |  | Residence | | |  | Residence | | |
| Metropolitan | 1,092,913 (54.75) | Reference |  | Metropolitan | 1,094,387 (54.74) | Reference |  | Metropolitan | 1,092,913 (54.75) | Reference |
| Others | 870,302 (43.60) | 1.12 (1.05–1.19) |  | Others | 871,658 (43.60) | 1.13 (1.08–1.19) |  | Others | 870,302 (43.60) | 1.12 (1.05–1.19) |
| Unknown | 32,959 (1.65) | 1.20 (0.96–1.50) |  | Unknown | 33,020 (1.65) | 1.22 (1.01–1.47) |  | Unknown | 32,959 (1.65) | 1.19 (0.95–1.49) |
| Income (percentile) | | |  | Income (percentile) | | |  | Income (percentile) | | |
| 17^th^–20^th^ | 295,168 (14.79) | Reference |  | 17^th^–20^th^ | 295,550 (14.78) | Reference |  | 17^th^–20^th^ | 295,168 (14.79) | Reference |
| 14^th^–16^th^ | 420,121 (21.05) | 0.94 (0.84–1.04) |  | 14^th^–16^th^ | 420,725 (21.05) | 1.00 (0.91–1.09) |  | 14^th^–16^th^ | 420,121 (21.05) | 0.94 (0.85–1.04) |
| 11^th^–13^th^ | 452,242 (22.66) | 1.03 (0.93–1.14) |  | 11^th^–13^th^ | 452,889 (22.66) | 1.04 (0.96–1.13) |  | 11^th^–13^th^ | 452,242 (22.66) | 1.03 (0.94–1.14) |
| 8^th^–10^th^ | 367,586 (18.41) | 0.93 (0.83–1.03) |  | 8^th^–10^th^ | 368,124 (18.41) | 0.98 (0.89–1.07) |  | 8^th^–10^th^ | 367,586 (18.41) | 0.93 (0.83–1.03) |
| 0–7^th^ | 417,207 (20.90) | 1.03 (0.93–1.14) |  | 0–7^th^ | 417,878 (20.90) | 1.06 (0.97–1.16) |  | 0–7^th^ | 417,207 (20.90) | 1.03 (0.93–1.14) |
| Unknown | 43,850 (2.20) | 1.04 (0.84–1.28) |  | Unknown | 43,899 (2.20) | 0.97 (0.80–1.16) |  | Unknown | 43,850 (2.20) | 1.04 (0.84–1.29) |
| Birth year | | |  | Birth year | | |  | Birth year | | |
| 2002 | 431,264 (21.60) | Reference |  | 2002 | 431,989 (21.61) | 1.00 (Reference) |  | 2002 | 431,264 (21.60) | Reference |
| 2003 | 426,789 (21.38) | 0.97 (0.89–1.06) |  | 2003 | 427,419 (21.38) | 0.95 (0.88–1.02) |  | 2003 | 426,789 (21.38) | 0.97 (0.89–1.06) |
| 2004 | 407,730 (20.43) | 1.05 (0.96–1.16) |  | 2004 | 408,321 (20.43) | 0.99 (0.92–1.07) |  | 2004 | 407,730 (20.43) | 1.06 (0.96–1.16) |
| 2005 | 372,142 (18.64) | 0.96 (0.87–1.07) |  | 2005 | 372,650 (18.64) | 0.91 (0.83–0.99) |  | 2005 | 372,142 (18.64) | 0.97 (0.87–1.08) |
| 2006 | 358,249 (17.95) | 0.95 (0.84–1.06) |  | 2006 | 358,686 (17.94) | 0.88 (0.80–0.96) |  | 2006 | 358,249 (17.95) | 0.96 (0.85–1.07) |

Statistically significant (p < 0.05) hazard ratios are indicated in bold. CI: confidence interval, URTI: upper respiratory tract infection, LRTI: lower respiratory tract infection, GTI: gastrointestinal tract infection.

**Table S5.** Hazard ratios for cancer incidence based on hospital visit frequency (outpatient visits only) for any infectious disease during three exposure windows (ages 0–4, 0–1, and 2–4 years).

| 0–4 years old | | |  | 0–1 years old | | |  | 2–4 years old | | |
| --- | --- | --- | --- | --- | --- | --- | --- | --- | --- | --- |
| Any infectious diseases, episodes | n (%) | Hazard ratio  (95% CI) |  | Any infectious diseases, episodes | n (%) | Hazard ratio  (95% CI) |  | Any infectious diseases, episodes | n (%) | Hazard ratio  (95% CI) |
| ≤4/year | 350,134 (17.54) | Reference |  | ≤4/year | 949,507 (47.50) | Reference |  | ≤4/year | 295,318 (14.79) | Reference |
| >4 & ≤5/year | 334,815 (16.77) | 1.05 (0.95–1.17) |  | >4 & ≤6/year | 650,486 (32.54) | 1.01 (0.96–1.07) |  | >4 & ≤6/year | 517,446 (25.92) | 1.10 (0.99–1.21) |
| >5 & ≤6/year | 468,534 (23.47) | 1.04 (0.94–1.14) |  | >6 & ≤8/year | 322,573 (16.14) | 1.03 (0.95–1.10) |  | **>6 & ≤7/year** | **394,090 (19.74)** | **1.11 (1.00**–**1.23)** |
| >6 & ≤8/year | 728,229 (36.48) | 1.08 (0.99–1.18) |  | >8/year | 76,499 (3.83) | 1.03 (0.90–1.18) |  | >7 & ≤8/year | 388,227 (19.45) | 1.08 (0.98–1.21) |
| >8 & ≤9/year | 95,158 (4.77) | 1.12 (0.97–1.30) |  |  |  |  |  | **>8 & ≤9/year** | **261,436 (13.10)** | **1.16 (1.04**–**1.30)** |
| >9/year | 19,304 (0.97) | 1.00 (0.74–1.37) |  |  |  |  |  | >9/year | 139,657 (7.00) | 1.13 (0.99–1.30) |

All models were adjusted for sex, residence, income, and birth year. Statistically significant (p < 0.05) hazard ratios are indicated in bold. CI: confidence interval.

**Table S6.** Hazard ratios for each cancer subtype incidence based on hospital visit frequency (outpatient visits or hospitalizations) for any infectious disease during three exposure windows (ages 0–4, 0–1, and 2–4 years).

| **0–4 years old** | |  | **0–1 years old** | |  | **2–4 years old** | |
| --- | --- | --- | --- | --- | --- | --- | --- |
| Infectious diseases, episodes | Hazard ratio  (95% CI) |  | Infectious diseases, episodes | Hazard ratio  (95% CI) |  | Infectious diseases, episodes | Hazard ratio  (95% CI) |
| Lymphoid and hematopoietic cancer |  |  | Lymphoid and hematopoietic cancer |  |  | Lymphoid and hematopoietic cancer |  |
| <4/year | Reference |  | ≤4/year | Reference |  | ≤4/year | Reference |
| ≥4 & <5/year | 1.04 (0.82–1.32) |  | >4 & ≤6/year | 1.01 (0.91–1.12) |  | >4 & ≤6/year | **1.23 (1.00–1.53)** |
| ≥5 & <6/year | 1.15 (0.93–1.41) |  | >6 & ≤8/year | 0.99 (0.89–1.11) |  | >6 & ≤7/year | 1.11 (0.89–1.38) |
| ≥6 & <8/year | 1.09 (0.91–1.32) |  | >8/year | 0.96 (0.81–1.14) |  | >7 & ≤8/year | 1.10 (0.89–1.35) |
| ≥8 & <9/year | 1.23 (0.98–1.54) |  |  |  |  | ≥8 & ≤9/year | 1.18 (0.95–1.46) |
| ≥9/year | 1.33 (0.97–1.82) |  |  |  |  | >9/year | **1.27 (1.02–1.59)** |
| Leukemia |  |  | Leukemia |  |  | Leukemia |  |
| <4/year | Reference |  | ≤4/year | Reference |  | ≤4/year | Reference |
| ≥4 & <5/year | 1.00 (0.73–1.36) |  | >4 & ≤6/year | 1.01 (0.89–1.15) |  | >4 & ≤6/year | 1.26 (0.95–1.68) |
| ≥5 & <6/year | 1.22 (0.93–1.60) |  | >6 & ≤8/year | 0.96 (0.83–1.11) |  | >6 & ≤7/year | 1.14 (0.86–1.53) |
| ≥6 & <8/year | 1.05 (0.82–1.35) |  | >8/year | 1.01 (0.82–1.25) |  | >7 & ≤8/year | 1.07 (0.81–1.42) |
| ≥8 & <9/year | 1.22 (0.91–1.65) |  |  |  |  | >8 & ≤9/year | 1.18 (0.89–1.57) |
| ≥9/year | 1.41 (0.94–2.12) |  |  |  |  | >9/year | 1.21 (0.90–1.63) |
| Lymphoid leukemia |  |  | Lymphoid leukemia |  |  | Lymphoid leukemia |  |
| <4/year | Reference |  | ≤4/year | Reference |  | ≤4/year | Reference |
| ≥4 & <5/year | 0.95 (0.65–1.40) |  | >4 & ≤6/year | 0.97 (0.83–1.13) |  | >4 & ≤6/year | 1.33 (0.94–1.89) |
| ≥5 & <6/year | 1.12 (0.81–1.56) |  | >6 & ≤8/year | 0.92 (0.77–1.10) |  | >6 & ≤7/year | 1.07 (0.75–1.54) |
| ≥6 & <8/year | 0.94 (0.70–1.27) |  | >8/year | 0.90 (0.70–1.18) |  | >7 & ≤8/year | 1.10 (0.77–1.56) |
| ≥8 & <9/year | 1.17 (0.81–1.67) |  |  |  |  | >8 & ≤9/year | 1.26 (0.88–1.78) |
| ≥9/year | 1.22 (0.73–2.05) |  |  |  |  | >9/year | 1.08 (0.74–1.57) |
| Myeloid leukemia |  |  | Myeloid leukemia |  |  | Myeloid leukemia |  |
| <4/year | Reference |  | ≤4/year | Reference |  | ≤4/year | Reference |
| ≥4 & <5/year | 0.87 (0.51–1.48) |  | >4 & ≤6/year | 1.21 (0.96–1.52) |  | >4 & ≤6/year | 1.14 (0.72–1.80) |
| ≥5 & <6/year | 1.23 (0.79–1.92) |  | >6 & ≤8/year | 1.03 (0.78–1.34) |  | >6 & ≤7/year | 1.06 (0.67–1.70) |
| ≥6 & <8/year | 1.02 (0.68–1.54) |  | >8/year | 1.01 (0.68–1.50) |  | >7 & ≤8/year | 1.06 (0.67–1.67) |
| ≥8 & <9/year | 1.05 (0.64–1.74) |  |  |  |  | >8 & ≤9/year | 0.97 (0.61–1.55) |
| ≥9/year | 1.19 (0.59–2.38) |  |  |  |  | >9/year | 1.10 (0.68–1.78) |
| Lymphoma |  |  | Lymphoma |  |  | Lymphoma |  |
| <4/year | Reference |  | ≤4/year | Reference |  | ≤4/year | Reference |
| ≥4 & <5/year | 1.07 (0.72–1.60) |  | >4 & ≤6/year | 0.90 (0.74–1.09) |  | >4 & ≤6/year | 1.11 (0.76–1.61) |
| ≥5 & <6/year | 1.00 (0.69–1.43) |  | >6 & ≤8/year | 1.01 (0.82–1.25) |  | >6 & ≤7/year | 1.07 (0.73–1.56) |
| ≥6 & <8/year | 1.03 (0.75–1.42) |  | >8/year | 0.86 (0.62–1.19) |  | >7 & ≤8/year | 1.11 (0.77–1.59) |
| ≥8 & <9/year | 1.01 (0.68–1.51) |  |  |  |  | >8 & ≤9/year | 1.13 (0.78–1.63) |
| ≥9/year | 1.26 (0.74–2.15) |  |  |  |  | >9/year | 1.10 (0.75–1.63) |
| Hodgkin-lymphoma |  |  | Hodgkin lymphoma |  |  | Hodgkin lymphoma |  |
| <4/year | Reference |  | ≤4/year | Reference |  | ≤4/year | Reference |
| ≥4 & <5/year | 0.36 (0.11–1.12) |  | >4 & ≤6/year | **0.53 (0.30–0.95)** |  | >4 & ≤6/year | **0.28 (0.11–0.77)** |
| ≥5 & <6/year | 0.50 (0.21–1.18) |  | >6 & ≤8/year | 0.67 (0.36–1.25) |  | >6 & ≤7/year | 0.66 (0.29–1.48) |
| ≥6 & <8/year | 0.73 (0.37–1.43) |  | >8/year | 0.82 (0.34–1.96) |  | >7 & ≤8/year | 0.74 (0.35–1.57) |
| ≥8 & <9/year | 0.33 (0.10–1.03) |  |  |  |  | >8 & ≤9/year | 0.64 (0.29–1.41) |
| ≥9/year | 0.27 (0.03–2.06) |  |  |  |  | >9/year | **0.28 (0.10–0.80)** |
| Non-Hodgkin lymphoma |  |  | Non-Hodgkin lymphoma |  |  | Non-Hodgkin lymphoma |  |
| <4/year | Reference |  | ≤4/year | Reference |  | ≤4/year | Reference |
| ≥4 & <5/year | 1.15 (0.76–1.74) |  | >4 & ≤6/year | 0.93 (0.76–1.14) |  | >4 & ≤6/year | 1.23 (0.83–1.82) |
| ≥5 & <6/year | 1.00 (0.68–1.45) |  | >6 & ≤8/year | 1.04 (0.83–1.29) |  | >6 & ≤7/year | 1.10 (0.73–1.64) |
| ≥6 & <8/year | 0.99 (0.71–1.39) |  | >8/year | 0.83 (0.59–1.17) |  | >7 & ≤8/year | 1.08 (0.73–1.59) |
| ≥8 & <9/year | 1.02 (0.68–1.55) |  |  |  |  | >8 & ≤9/year | 1.13 (0.76–1.68) |
| ≥9/year | 1.31 (0.75–2.28) |  |  |  |  | >9/year | 1.21 (0.81–1.82) |
| CNS cancer |  |  | CNS cancer |  |  | CNS cancer |  |
| <4/year | Reference |  | ≤4/year | Reference |  | ≤4/year | Reference |
| ≥4 & <5/year | 0.90 (0.65–1.25) |  | >4 & ≤6/year | 1.03 (0.88–1.19) |  | >4 & ≤6/year | 0.93 (0.70–1.25) |
| ≥5 & <6/year | 1.08 (0.81–1.424) |  | >6 & ≤8/year | 1.06 (0.90–1.25) |  | >6 & ≤7/year | 1.12 (0.84–1.49) |
| ≥6 & <8/year | 1.08 (0.85–1.39) |  | >8/year | 1.12 (0.88–1.42) |  | >7 & ≤8/year | 1.12 (0.85–1.48) |
| ≥8 & <9/year | 1.05 (0.77–1.43) |  |  |  |  | >8 & ≤9/year | 0.99 (0.74–1.31) |
| ≥9/year | 1.00 (0.63–1.58) |  |  |  |  | >9/year | 1.09 (0.81–1.47) |
| Brain cancer |  |  | Brain cancer |  |  | Brain cancer |  |
| <4/year | Reference |  | ≤4/year | Reference |  | ≤4/year | Reference |
| ≥4 & <5/year | 0.91 (0.66–1.27) |  | >4 & ≤6/year | 1.00 (0.86–1.17) |  | >4 & ≤6/year | 0.92 (0.68–1.23) |
| ≥5 & <6/year | 1.05 (0.79–1.39) |  | >6 & ≤8/year | 1.08 (0.91–1.28) |  | >6 & ≤7/year | 1.12 (0.84–1.50) |
| ≥6 & <8/year | 1.07 (0.83–1.38) |  | >8/year | 1.14 (0.89–1.47) |  | >7 & ≤8/year | 1.12 (0.85–1.49) |
| ≥8 & <9/year | 1.04 (0.76–1.43) |  |  |  |  | >8 & ≤9/year | 0.95 (0.71–1.28) |
| ≥9/year | 0.98 (0.62–1.57) |  |  |  |  | >9/year | 1.09 (0.81–1.47) |
| Bone cancer |  |  | Bone cancer |  |  | Bone cancer |  |
| <4/year | Reference |  | ≤4/year | Reference |  | ≤4/year | Reference |
| ≥4 & <5/year | 0.99 (0.61–1.60) |  | >4 & ≤6/year | 1.08 (0.86–1.36) |  | >4 & ≤6/year | 1.06 (0.69–1.64) |
| ≥5 & <6/year | 1.20 (0.80–1.82) |  | >6 & ≤8/year | 1.09 (0.84–1.41) |  | >6 & ≤7/year | 1.12 (0.72–1.73) |
| ≥6 & <8/year | 1.04 (0.71–1.51) |  | >8/year | 0.89 (0.59–1.34) |  | >7 & ≤8/year | 1.29 (0.85–1.96) |
| ≥8 & <9/year | 0.81 (0.49–1.34) |  |  |  |  | >8 & ≤9/year | 0.93 (0.60–1.45) |
| ≥9/year | 0.67 (0.30–1.52) |  |  |  |  | >9/year | 0.74 (0.45–1.20) |
| Soft tissue sarcoma |  |  | Soft tissue sarcoma |  |  | Soft tissue sarcoma |  |
| <4/year | Reference |  | ≤4/year | Reference |  | ≤4/year | Reference |
| ≥4 & <5/year | 1.72 (0.89–3.31) |  | >4 & ≤6/year | 1.02 (0.77–1.34) |  | >4 & ≤6/year | 1.93 (0.99–3.75) |
| ≥5 & <6/year | 1.46 (0.79–2.71) |  | >6 & ≤8/year | 1.03 (0.76–1.41) |  | >6 & ≤7/year | 1.16 (0.57–2.38) |
| ≥6 & <8/year | 1.51 (0.86–2.65) |  | >8/year | **1.51 (1.02–2.25)** |  | >7 & ≤8/year | 1.89 (0.98–3.65) |
| ≥8 & <9/year | 1.41 (0.71–2.78) |  |  |  |  | >8 & ≤9/year | **2.39 (1.25–4.58)** |
| ≥9/year | **3.01 (1.41–6.43)** |  |  |  |  | >9/year | 1.81 (0.91–3.61) |
| Renal cancer |  |  | Renal cancer |  |  | Renal cancer |  |
| <4/year | Reference |  | ≤4/year | Reference |  | ≤4/year | Reference |
| ≥4 & <5/year | 1.11 (0.48–2.58) |  | >4 & ≤6/year | 0.85 (0.59–1.22) |  | >4 & ≤6/year | 1.33 (0.59–2.99) |
| ≥5 & <6/year | 1.01 (0.47–2.17) |  | >6 & ≤8/year | 0.80 (0.53–1.21) |  | >6 & ≤7/year | 0.87 (0.36–2.07) |
| ≥6 & <8/year | 0.88 (0.44–1.75) |  | >8/year | 0.98 (0.55–1.73) |  | >7 & ≤8/year | 0.95 (0.41–2.17) |
| ≥8 & <9/year | 0.97 (0.41–2.30) |  |  |  |  | >8 & ≤9/year | 1.33 (0.59–2.99) |
| ≥9/year | 1.60 (0.54–4.69) |  |  |  |  | >9/year | 1.27 (0.54–2.97) |
| Liver cancer |  |  | Liver cancer |  |  | Liver cancer |  |
| <4/year | Reference |  | ≤4/year | Reference |  | ≤4/year | Reference |
| ≥4 & <5/year | 0.80 (0.30–2.15) |  | >4 & ≤6/year | 1.03 (0.68–1.56) |  | >4 & ≤6/year | 0.84 (0.33–2.18) |
| ≥5 & <6/year | 0.46 (0.17–1.25) |  | >6 & ≤8/year | 0.88 (0.54–1.43) |  | >6 & ≤7/year | 1.00 (0.39–2.56) |
| ≥6 & <8/year | 1.10 (0.53–2.28) |  | >8/year | 0.93 (0.45–1.90) |  | >7 & ≤8/year | 1.02 (0.41–2.52) |
| ≥8 & <9/year | 1.18 (0.49–2.86) |  |  |  |  | >8 & ≤9/year | 1.06 (0.43–2.65) |
| ≥9/year | 0.35 (0.04–2.78) |  |  |  |  | >9/year | 1.66 (0.69–4.00) |
| Other cancer |  |  | Other cancer |  |  | Other cancer |  |
| <4/year | Reference |  | ≤4/year | Reference |  | ≤4/year | Reference |
| ≥4 & <5/year | 1.16 (0.91–1.48) |  | >4 & ≤6/year | **1.14 (1.03–1.27)** |  | >4 & ≤6/year | 1.12 (0.90–1.39) |
| ≥5 & <6/year | **1.25 (1.01–1.55)** |  | >6 & ≤8/year | 1.08 (0.96–1.21) |  | >6 & ≤7/year | 1.05 (0.84–1.31) |
| ≥6 & <8/year | **1.29 (1.06–1.57)** |  | >8/year | 1.03 (0.86–1.23) |  | >7 & ≤8/year | 1.19 (0.96–1.47) |
| ≥8 & <9/year | **1.41 (1.12–1.79)** |  |  |  |  | >8 & ≤9/year | 1.21 (0.98–1.50) |
| ≥9/year | 0.97 (0.67–1.42) |  |  |  |  | >9/year | 1.17 (0.93–1.46) |

All models were adjusted for sex, residence, income, and birth year. Statistically significant (p < 0.05) hazard ratios are indicated in bold. CI: confidence interval.

**Table S7.** Hazard ratios for each cancer subtype incidence based on hospital visit frequency (hospitalizations only) for any infectious disease during the three exposure windows (ages 0–4, 0–1, and 2–4 years).

| **0–4 years old** | |  | **0–1 years old** | |  | **2–4 years old** | |
| --- | --- | --- | --- | --- | --- | --- | --- |
| Infectious diseases,  episodes | Hazard ratio  (95% CI) |  | Infectious diseases,  episodes | Hazard ratio  (95% CI) |  | Infectious diseases,  episodes | Hazard Ratio  (95% CI) |
| Lymphoid and hematopoietic cancer |  |  | Lymphoid and hematopoietic cancer |  |  | Lymphoid and hematopoietic cancer |  |
| 0 | Reference |  | 0 | Reference |  | 0 | Reference |
| 1 | 1.07 (0.96–1.20) |  | 1 | 1.05 (0.93–1.18) |  | 1 | **1.15 (1.01–1.31)** |
| 2 | 1.20 (0.99–1.45) |  | ≥2 | 1.24 (0.98–1.57) |  | ≥2 | 1.15 (0.92–1.45) |
| ≥3 | **1.47 (1.21–1.78)** |  |  |  |  |  |  |
| Leukemia |  |  | Leukemia |  |  | Leukemia |  |
| 0 | Reference |  | 0 | Reference |  | 0 | Reference |
| 1 | 0.97 (0.83–1.13) |  | 1 | 0.96 (0.82–1.12) |  | 1 | 1.00 (0.84–1.20) |
| 2 | 1.14 (0.89–1.47) |  | ≥2 | 1.10 (0.80–1.50) |  | ≥2 | 1.12 (0.83–1.51) |
| ≥3 | 1.09 (0.82–1.45) |  |  |  |  |  |  |
| Lymphoid leukemia |  |  | Lymphoid leukemia |  |  | Lymphoid leukemia |  |
| 0 | Reference |  | 0 | Reference |  | 0 | Reference |
| 1 | 0.97 (0.80–1.17) |  | 1 | 0.97 (0.81–1.17) |  | 1 | 1.08 (0.87–1.35) |
| 2 | **1.35 (1.00–1.81)** |  | ≥2 | 1.17 (0.82–1.68) |  | ≥2 | 0.90 (0.59–1.36) |
| ≥3 | 0.92 (0.63–1.35) |  |  |  |  |  |  |
| Myeloid leukemia |  |  | Myeloid leukemia |  |  | Myeloid leukemia |  |
| 0 | Reference |  | 0 | Reference |  | 0 | Reference |
| 1 | 0.85 (0.65–1.10) |  | 1 | 1.02 (0.77–1.34) |  | 1 | 0.75 (0.54–1.05) |
| 2 | 0.91 (0.58–1.45) |  | ≥2 | 0.75 (0.37–1.51) |  | ≥2 | 1.09 (0.66–1.82) |
| ≥3 | 0.98 (0.60–1.61) |  |  |  |  |  |  |
| Lymphoma |  |  | Lymphoma |  |  | Lymphoma |  |
| 0 | Reference |  | 0 | Reference |  | 0 | Reference |
| 1 | 1.17 (0.96–1.43) |  | 1 | 1.08 (0.87–1.35) |  | 1 | 1.23 (0.98–1.53) |
| 2 | 1.23 (0.88–1.73) |  | ≥2 | **1.62 (1.09–2.40)** |  | ≥2 | 0.98 (0.64–1.51) |
| ≥3 | 1.29 (0.89–1.86) |  |  |  |  |  |  |
| Hodgkin lymphoma |  |  | Hodgkin lymphoma |  |  | Hodgkin lymphoma |  |
| 0 | Reference |  | 0 | Reference |  | 0 | Reference |
| 1 | 0.88 (0.49–1.58) |  | 1 | 0.90 (0.45–1.82) |  | 1 | 0.89 (0.45–1.76) |
| 2 | 1.41 (0.58–3.42) |  | ≥2 | 1.75 (0.54–5.72) |  | ≥2 | 1.65 (0.65–4.19) |
| ≥3 | 1.99 (0.86–4.60) |  |  |  |  |  |  |
| Non-Hodgkin lymphoma |  |  | Non-Hodgkin lymphoma |  |  | Non-Hodgkin lymphoma |  |
| 0 | Reference |  | 0 | Reference |  | 0 | Reference |
| 1 | **1.24 (1.01–1.53)** |  | 1 | 1.10 (0.88–1.38) |  | 1 | 1.25 (0.99–1.58) |
| 2 | 1.31 (0.92–1.86) |  | ≥2 | **1.54 (1.02–2.34)** |  | ≥2 | 0.98 (0.62–1.55) |
| ≥3 | 1.23 (0.82–1.83) |  |  |  |  |  |  |
| CNS cancer |  |  | CNS cancer |  |  | CNS cancer |  |
| 0 | Reference |  | 0 | Reference |  | 0 | Reference |
| 1 | 1.20 (1.03–1.41) |  | 1 | 1.11 (0.94–1.32) |  | 1 | 1.14 (0.95–1.36) |
| 2 | 1.16 (0.88–1.55) |  | ≥2 | 1.16 (0.80–1.68) |  | ≥2 | 1.05 (0.75–1.47) |
| ≥3 | 1.41 (1.05–1.88) |  |  |  |  |  |  |
| Brain cancer |  |  | Brain cancer |  |  | Brain cancer |  |
| 0 | Reference |  | 0 | Reference |  | 0 | Reference |
| 1 | **1.21 (1.03–1.42)** |  | 1 | 1.13 (0.95–1.35) |  | 1 | 1.11 (0.92–1.34) |
| 2 | 1.21 (0.91–1.61) |  | ≥2 | 1.25 (0.86–1.81) |  | ≥2 | 1.06 (0.76–1.49) |
| ≥3 | **1.36 (1.01–1.83)** |  |  |  |  |  |  |
| Bone cancer |  |  | Bone cancer |  |  | Bone cancer |  |
| 0 | Reference |  | 0 | Reference |  | 0 | Reference |
| 1 | 0.99 (0.77–1.27) |  | 1 | 1.06 (0.81–1.39) |  | 1 | 1.21 (0.92–1.59) |
| 2 | 1.29 (0.87–1.92) |  | ≥2 | 1.29 (0.75–2.21) |  | ≥2 | 0.91 (0.53–1.56) |
| ≥3 | 1.11 (0.70–1.76) |  |  |  |  |  |  |
| Soft tissue sarcoma |  |  | Soft tissue sarcoma |  |  | Soft tissue sarcoma |  |
| 0 | Reference |  | 0 | Reference |  | 0 | Reference |
| 1 | 0.85 (0.62–1.18) |  | 1 | 1.34 (0.99–1.80) |  | 1 | 0.95 (0.65–1.38) |
| 2 | 1.20 (0.72–2.00) |  | ≥2 | 1.24 (0.63–2.43) |  | ≥2 | 1.21 (0.67–2.18) |
| ≥3 | 1.05 (0.59–1.89) |  |  |  |  |  |  |
| Renal cancer |  |  | Renal cancer |  |  | Renal cancer |  |
| 0 | Reference |  | 0 | Reference |  | 0 | Reference |
| 1 | 0.98 (0.63–1.54) |  | 1 | 0.87 (0.56–1.36) |  | 1 | 1.31 (0.80–2.13) |
| 2 | 1.46 (0.75–2.84) |  | ≥2 | 0.56 (0.18–1.77) |  | ≥2 | 1.65 (0.79–3.45) |
| ≥3 | 0.79 (0.31–2.00) |  |  |  |  |  |  |
| Liver cancer |  |  | Liver cancer |  |  | Liver cancer |  |
| 0 | Reference |  | 0 | Reference |  | 0 | Reference |
| 1 | 0.95 (0.56–1.59) |  | 1 | 1.17 (0.72–1.90) |  | 1 | 0.51 (0.23–1.11) |
| 2 | 0.64 (2.00–2.08) |  | ≥2 | 1.40 (0.51–3.85) |  | ≥2 | 1.39 (0.55–3.49) |
| ≥3 | 1.81 (0.80–4.10) |  |  |  |  |  |  |
| Other cancer |  |  | Other cancer |  |  | Other cancer |  |
| 0 | Reference |  | 0 | Reference |  | 0 | Reference |
| 1 | 1.03 (0.92–1.17) |  | 1 | 1.02 (0.90–1.16) |  | 1 | 1.14 (0.99–1.30) |
| 2 | 1.15 (0.93–1.42) |  | ≥2 | **1.28 (1.00–1.65)** |  | ≥2 | **1.32 (1.04–1.67)** |
| ≥3 | 1.21 (0.96–1.53) |  |  |  |  |  |  |

All models were adjusted for sex, residence, income, and birth year. Statistically significant (p < 0.05) hazard ratios are indicated in bold. CI: confidence interval.
